# Machine Learning Models to Identify Clinically Significant Anxiety in Short-Term Insomnia Using Accelerometers

**DOI:** 10.1155/da/3082856

**Published:** 2025-05-13

**Authors:** Leqin Fang, Weixiong Zeng, Shuqiong Zheng, Shixu Du, Hangyi Yang, Xue Luo, Shufei Zeng, Zhiting Huang, Weiguo Chen, Bin Zhang

**Affiliations:** ^1^Department of Psychiatry, Sleep Medicine Center, Nanfang Hospital, Southern Medical University, Guangzhou, China; ^2^Institute of Brain Disease, Nanfang Hospital, Southern Medical University, Guangzhou, China; ^3^Key Laboratory of Mental Health of the Ministry of Education, Southern Medical University, Guangzhou, China; ^4^Department of Radiology, Nanfang Hospital, Southern Medical University, Guangzhou, China

**Keywords:** accelerometer, circadian rhythm, clinically significant anxiety, machine learning, physical activity, short-term insomnia

## Abstract

Clinically significant anxiety (CSA) is common in individuals with short-term insomnia. This study aims to explore the relationship between CSA and the subjective and objective parameters of sleep in patients with short-term insomnia and construct machine learning (ML) models to determine the utility of accelerometer features in identifying significant anxiety. A total of 205 short-term insomnia participants from China were assigned to the group with CSA (*N* = 33) or the group without CSA (*N* = 172). Interaction analysis based on linear regression was used to estimate the possible interactive effect of accelerometer features between CSA and sleep problems. Four feature sets and eight algorithms were used to construct ML models, with Shapley Additive exPlanations (SHAP) values used to visualize feature importance and influence processes. CSA in patients with short-term insomnia leads to more severe subjective sleep problems, and accelerometer-measured features warrant further attention for the identification of interactive factors. A significant interaction effect was found between anxiety symptoms and longer duration of physical activity on insomnia severity (*P*_interaction_ < 0.05). Anxiety symptoms and interdaily stability had an interactive association with sleep hygiene behaviors (*P*_interaction_ < 0.01). ML can process and analyze complex accelerometer features to identify CSA in patients with short-term insomnia. Compared with other feature sets and algorithms, the XGBoost model with accelerometer-measured features on weekdays more effectively identified CSA with area under the curve (AUC) value of 0.777. SHAP analysis results indicated that circadian rhythm features had significant contributions. Decision plots based on SHAP were applied to visualize the personalized risk factors for each patient and provide clinicians with more easily understandable and practical explanation methods that enhance clinical decision-making.

**Trial Registration:** Chinese Clinical Trial Registry identifier: ChiCTR2200062910

## 1. Introduction

Short-term insomnia is defined as a sleep disturbance that persists for less than 3 months and meets the International Classification of Sleep Disorders, Third Edition, Text Revision (ICSD-3-TR) criteria [1]. Spielman et al. [2] reported that short-term insomnia is the most intense type of insomnia, with its severity gradually declining over time. Short-term insomnia represents a critical transitional stage toward chronic insomnia, often accompanied by anxiety symptoms [3]. When anxiety symptoms progress to moderate or severe levels, they are classified as clinically significant anxiety (CSA), which often requires clinical intervention [4]. Recently, our team reported that CSA negatively affects the effectiveness of treatments for short-term insomnia [5]. CSA may exacerbate sleep pattern (SP) disruption [6], sleep-related beliefs, attitudes, and behaviors, increasing the risk of short-term insomnia progressing to chronic insomnia [7]. CSA is not only a predictive factor for short-term insomnia but also a major contributor to the persistence or worsening of insomnia. Recognizing this is essential for developing personalized treatment strategies [7]. However, research exploring how CSA specifically affects subjective and objective parameters of sleep in short-term insomnia is lacking [6, 8].

Subjective self-report questionnaires are commonly used to assess CSA [9]. However, CSA can affect the cognition and behavior of individuals with short-term insomnia, presenting a challenge in the accurate and objective monitoring of CSA in daily life [10, 11]. This suggests the necessity of utilizing objective measurements for the recognition of CSA. Accelerometers provide a clinically viable and quantifiable objective measure that is commonly used for tracking SPs, physical activity (PA), and circadian rhythms (CRs) [6, 12]. Previous studies have shown that individuals with anxiety symptoms have significantly poorer sleep efficiency (SE) and greater time spent awake after sleep onset, low PA levels, and daily rhythm disturbances based on accelerometer findings [6, 13]. Therefore, accelerometers have the potential to enhance the utility of subjective measures of insomnia by providing objective data on SPs, CRs, and PA [14], and the promise of using these devices to objectively monitor CSA is encouraging.

The abundant accelerometer data collected, which integrate SP, PA, and CR information coupled with machine learning (ML), can help demonstrate the relationship and predict symptom dynamics in a much more cost-effective way [15]. ML is a subfield of artificial intelligence that can process integrated information and analyze complex relationships in data [16, 17]. However, current research on processing accelerometer data with ML to monitor and predict CSA remains insufficient. Jacobson reported that ML can effectively utilize accelerometer data to accurately predict the risk of anxiety [18]. However, these findings may have been impacted by potential sleep disorders.

Thus, in this study, we aimed to determine the relationship between CSA and the subjective and objective parameters of sleep in short-term insomnia and test the viability of building CSA predictive models using ML methods and accelerometer data. On the basis of prior related studies, we propose the following hypothesis:

Hypothesis 1: CSA in short-term insomnia is associated with worse performance in both subjective and objective sleep measurements.

Hypothesis 2: Different periods of accelerometer-measured features (weekday, weekend and total periods) can accurately predict CSA in short-term insomnia patients using ML.

## 2. Method

### 2.1. Study Design and Participants

**Figure 1:**
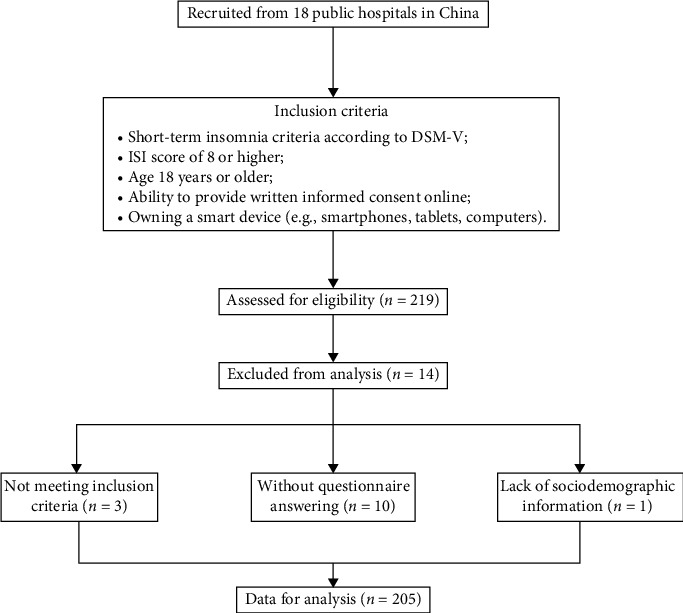
Flowchart of the study.

This study is a secondary analysis of a multicenter clinical trial involving 219 participants with short-term insomnia (registered at ClinicalTrials.gov, ID: ChiCTR2200062910, ethical approval ref: NFEC-202204-K14). The participants were recruited through advertisements. All eligible participants were recruited through the following procedure. First, clinical diagnosis and evaluation were conducted by physicians. Second, participants who met the diagnostic criteria completed a structured clinical interview for further screening, which was conducted by research staff who had undergone standardized training. The inclusion criteria were as follows: (1) short-term insomnia according to the DSM-V; (2) insomnia severity index (ISI) score of 8 or higher; (3) age 18 years or older; (4) ability to provide written informed consent; and (5) owning a smart device (e.g., smartphones, tablets, or computers). The exclusion criteria included (1) participants with a clinical diagnosis of unstable physical diseases (e.g., heart failure, severe asthma, severe pneumonia) or mental disorders (e.g., depression, bipolar disorder, schizophrenia); (2) other sleep disorders (e.g., obstructive sleep apnea and restless legs syndrome) diagnosed using polysomnographic findings; (3) shift workers or frequent flyers crossing time zones; and (4) pregnant or breastfeeding women. The flowchart of this study is presented in Figure 1. Of the 219 participants who provided baseline accelerometer data, 14 participants were removed because of a lack of questionnaire data or sociodemographic information. Finally, 205 participants were included in this study.

### 2.2. Accelerometer-Measured Feature Processing

Accelerometer-measured features were assessed using a wrist-worn triaxial accelerometer (GENEActive, Activinsights Lts, UK). The accelerometers were set to record with a 100 Hz sampling frequency and were worn on the participants' non-dominant wrists. The accelerometers were used to collect data for a minimum of consecutive 7-day periods, including weekdays and weekends [19, 20]. The data were analyzed via the R packages GGIR (v-2.4-0, https://cran.r-project.org/web/packages/GGIR/index.html) and postGGIR (V.2.4.0.2, https://cran.r-project.org/web/packages/postGGIR/index.html). The outcomes of the accelerometer-measured features were divided into three parts: features on weekdays, weekends, and 7-day periods.

### 2.3. Assessment of CSA

The severity of anxiety symptoms was measured using the Generalized Anxiety Disorder 7-item scale (GAD-7, scale 0–3, range 0–21, with higher scores indicating more severe anxiety) [21]. Scores ≥ 10 are regarded as CSA [22]. The participants were divided into two groups according to their GAD-7 scores: short-term insomnia with CSA (GAD-7 score ≥ 10, *N* = 33) and short-term insomnia without CSA (GAD-7 score < 10, *N* = 172).

### 2.4. Outcomes

The severity of insomnia symptoms was measured using the ISI (scale 0–4; range 0–28) [23]. The severity of daytime sleepiness was measured using the Epworth Sleepiness Scale (ESS, scale 0–3; range 0–24) [24]. The level of presleep arousal was measured using the presleep arousal scale (PSAS, scale 1–5; range 16–80) [25]. Sleep reactivity (probability of stress-induced sleep disturbances) was measured using the Ford Insomnia Response to Stress Test (FIRST, scale 1–4; range 9–36) [26]. Sleep hygiene behaviors were measured using the Sleep Hygiene Practice Scale (SHPS, scale 1–6; range 30–180) [27]. Sleep-related beliefs and attitudes were measured using the 30-item Dysfunctional Beliefs and Attitudes about Sleep (DBAS, scale 1–5; range 30–150) [28]. Sleep effort, which refers to both cognitive and behavioural conscious attempts to initiate sleep, were measured using the Glasgow Sleep Effort Scale (GSES, scale 0–2; range 0–14) [29]. The severity of depressive symptoms was measured using the Patient Health Questionnaire 9-item (PHQ-9, scale 0–3, range 0–27) [30]. Total sleep time (TST), SE, relative amplitude (RA), interdaily stability (IS), intradaily variability (IV), sedentary times, light activity times and moderate and vigorous PA times (MVPAs) were measured using the accelerometer. A description of the accelerometer-measured outcomes is provided in Supporting Information Table S1.

### 2.5. Data Preprocessing for ML

Four datasets were constructed, including the previously described records of accelerometer features on weekdays, weekends, and 7-day periods, and the merged accelerometer features of all three datasets. The mean calculated from the complete dataset was used to impute the missing data. The proportion of missing values for the features is less than 8%, and the report of missing values is presented in Supporting Information Table S2. The datasets were divided into training and validation sets at a 7 : 3 ratio using random stratified sampling. Quantitative data were normalized to a range of 0–1 to standardize the data dimensions, thereby accelerating and improving model training [31]. To address the issue of data sample imbalance, we used the synthetic minority oversampling technique (SMOTE) to balance the training set. This method increases the number of minority class samples by generating synthetic samples, thereby achieving data balance and improving model training effectiveness [32]. The SMOTE algorithm was implemented using the Imblearn Python package.

### 2.6. ML Model Construction

To avoid potential bias arising from the limitations of a single model, we chose to comprehensively compare eight commonly used and high-performing ML algorithms: extreme gradient boosting (XGBoost), decision tree (DT), random forest (RF), support vector machine (SVM), *k*-nearest neighbor (KNN), adaptive boosting (AdaBoost), multilayer perceptron (MLP), and naive Bayes (NB). We implemented these algorithms in Python using the scikit-learn and XGBoost packages to predict CSA [33]. We trained the models using 10-fold cross-validation and employed the grid search method to traverse the predefined parameter combinations to retrieve the optimal hyperparameters. After completing the model training, we validated the classification performance of these ML models on an independent validation set. The model evaluation metrics included the area under the receiver operating characteristic curve (AUC), sensitivity, specificity, accuracy, recall, positive predictive value (PPV), negative predictive value (NPV), F1 score, Youden index, and decision curve analysis (DCA). For detailed definitions and calculation formulas of these metrics, please refer to Supporting Information S1.

### 2.7. Model Explanation

We added the locally explanatory technique Shapley Additive exPlanations (SHAP) to visualize the results of feature importance and contribution. SHAP is commonly employed as a robust approach derived from cooperative game theory. This technique explains the decision process of ML models by calculating the relative contribution of each feature, allowing for a deeper understanding of how individual features impact model predictions. By attributing each feature's contribution to the final prediction, SHAP helps in identifying and interpreting key factors that influence model behavior [16]. We used the Python SHAP package to compute SHAP values for each feature in the optimal model and plotted the corresponding feature contribution graphs, identifying the accelerometer features with the greatest impact on predicting CSA.

### 2.8. Statistical Analysis

Data from accelerometers with at least 5 days, including at least 1 weekend day of ≥ 90% of 24 h (21.6 h) of wear time on each of the days, were considered valid [34]. Accelerometer-measured features were averaged across a monitoring period. Descriptive statistics were calculated for all metrics (means ± SDs) or medians (twentyfifth to seventyfifth percentiles) following normality testing. The proportion and pattern of missing data were analyzed. All analyses were conducted with complete cases.

To evaluate the joint associations between CR- or PA-related variables and CSA in short-term insomnia, we derived a combined accelerometer-measured feature and CSA grouping variable with four distinct categories according to the combination of above/below median values for RA (0.80) between day-time and night-time activity, IS (0.35), interdaily variability (IV, 0.80), sedentary times (792.62 min/day), moderate and vigorous PA times (MVPA, 65.50 min/day), and CSA. Linear regression models were used to examine the association of each accelerometer-measured feature/CSA combination and were adjusted for covariates. The potential covariates included age, sex, education, body mass index (BMI), marital status, and job. The interaction between accelerometer-measured features and CSA was also examined by including an interaction term within the regression models.

All the statistical analyses were performed with R Statistical Software (R Foundation for Statistical Computing, Vienna, Austria). A *p* value of < 0.05 was considered to indicate statistical significance.

The AUC values of the ML models were compared using the DeLong test. The sample size was determined on the basis of a significance test for the incidence of CSA (*α* = 0.05, 1-*β* = 0.8, *power* = 0.8) using MedCalc 19.0.7 (MedCalc Software Ltd., Ostend, Belgium). Further details are provided in Supporting Information Figure S1.

## 3. Results

### 3.1. Characteristics of the Study Participants

Table 1 presents the characteristics of the remaining 205 short-term insomnia participants. Compared with the group without CSA, the group with CSA consisted predominantly of women. The group with CSA had higher scores on the ISI, FIRST, GSES, SHPS, PSAS, and PHQ and lower scores on the DBAS, suggesting poorer subjective sleep quality. For accelerometer-measured features, short-term insomnia participants with CSA experienced a longer TST. Additionally, they also reported a longer duration of sedentary activity, a shorter duration of MVPA, and a higher level of RA, indicating a greater distinction between activity levels during the most or least active periods of the day.

### 3.2. Joint Associations of Accelerometer-Measured Features and CSA With Subjective Insomnia Outcomes

**Figure 2:**
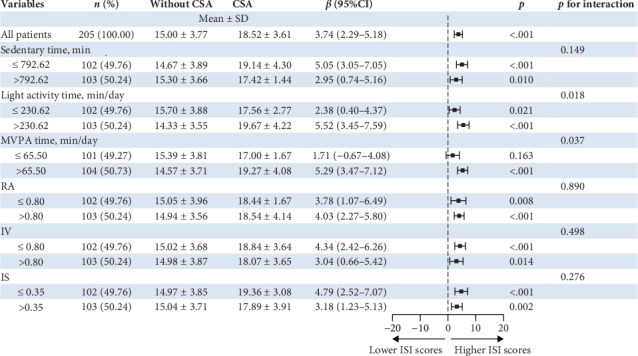
Forest plot estimating the ISI scores according to joint associations of accelerometer-measured features and CSA. *β* and 95% confidence intervals were weighted to be nationally representative. Linear regression models were adjusted for age, sex, education, BMI, marriage, education, and job. Sedentary time was defined as low (≤792.62 min/day) or high (>792.62 min/day) according to the sample median. The light activity time was defined as low (≤230.62 min/day) or high (>230.62 min/day). MVPA time was defined as low (≤65.50 min/day) or high (>65.50 min/day) according to the sample median. RA was defined as low (≤0.80) or high (>0.80) according to the sample median. IV was defined as low (≤0.80) or high (>0.80) according to the sample median. IS was defined as low (≤0.35) or high (>0.35) according to the sample median. CSA, clinically significant anxiety; IS, interdaily stability; IV, intradaily variability; MVPA, moderate to vigorous physical activity; RA, relative amplitude.

Figure 2 shows the joint associations of accelerometer-measured features and CSA with ISI scores among short-term insomnia participants. In relation to the PA features, for the light activity time *⁣*^*∗*^ CSA and the MVPA time *⁣*^*∗*^ CSA, the interaction *P* values were 0.018 and 0.037 for the ISI scores, respectively. The CSA group was associated with higher ISI scores among individuals with high light activity times (>230.62 min/day) and MVPA times (>65.50 min/day). However, for those with low light activity durations (≤230.62 min/day), CSA was less strongly associated with higher ISI scores. Among participants who engaged in lower MVPA time, CSA was not associated with higher ISI scores. In relation to the CR features, the RA *⁣*^*∗*^ CSA, IV *⁣*^*∗*^ CSA, and IS *⁣*^*∗*^CSA were not significant for the interaction based on *p* values (*P*_interaction_ > 0.05).

**Figure 3:**
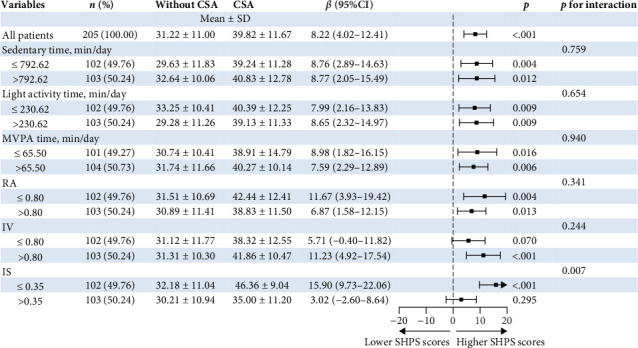
Forest plot estimating the SHPS scores according to joint associations of accelerometer-measured features and CSA. *β* and 95% confidence intervals were weighted to be nationally representative. Linear regression models were adjusted for age, sex, education, BMI, marriage, education, and job. Sedentary time was defined as low (≤792.62 min/day) or high (>792.62 min/day) according to the sample median. The light activity time was defined as low (≤230.62 min/day) or high (>230.62 min/day). MVPA time was defined as low (≤65.50 min/day) or high (>65.50 min/day) according to the sample median. RA was defined as low (≤0.80) or high (>0.80) according to the sample median. IV was defined as low (≤0.80) or high (>0.80) according to the sample median. IS was defined as low (≤0.35) or high (>0.35) according to the sample median. CSA, clinically significant anxiety; MVPA, moderate to vigorous physical activity; RA, relative amplitude; IV intradaily variability; IS, interdaily stability.

Figure 3 shows the joint associations of accelerometer-measured features and CSA with SHPS scores among short-term insomnia participants. In relation to the CR features, the IS *⁣*^*∗*^ CSA interaction *p* value was 0.007 for the SHPS scores. Compared with the group without CSA, the CSA group was associated with higher SHPS scores among participants with greater IS (>0.35). Among short-term insomnia participants who engaged in lower IS (≤0.35), the group with CSA was not significantly associated with higher SHPS scores. In relation to PA features, MVPA time *⁣*^*∗*^ CSA and sedentary time *⁣*^*∗*^ CSA were not significant for interaction *p* values (*P*_interaction_ >0.05).

We did not find joint associations of accelerometer-measured features and anxiety with other sleep outcomes, including FIRST scores (*P*_interaction_ > 0.05, Supporting Information Figure S2), GSES scores (*P*_interaction_ > 0.05, Supporting Information Figure S3), DBAS scores (*P*_interaction_ > 0.05, Supporting Information Figure S4), PSAS scores (*P*_interaction_ > 0.05, Supporting Information Figure S5), MEQ scores (*P*_interaction_ > 0.05, Supporting Information Figure S6), ESS scores (*P*_interaction_ > 0.05, Supporting Information Figure S7), TST (*P*_interaction_ > 0.05, Supporting Information Figure S8), and SE (*P*_interaction_ > 0.05, Supporting Information Figure S9).

### 3.3. Model Performance

**Figure 4:**
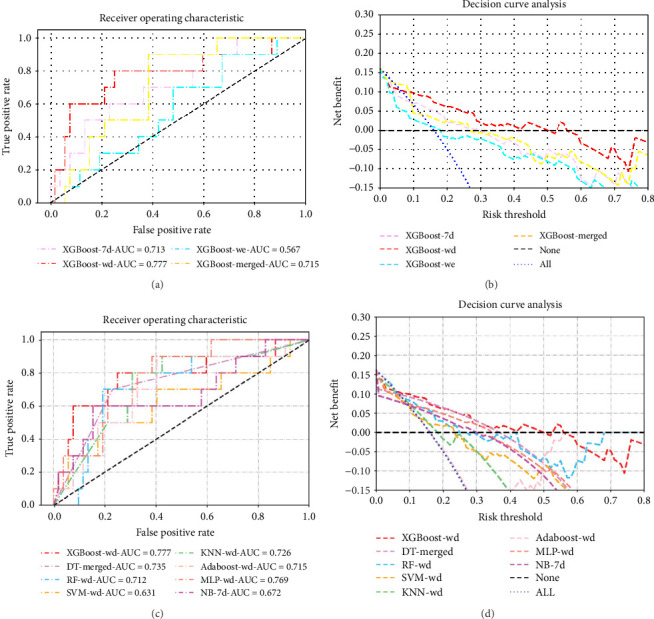
The results from the ML models identifying CSA. (a, b) ROC curves and DCA results for the XGBoost models constructed on the four feature sets. (c, d) ROC curves and DCA for the optimal model in each ML algorithm. CSA, clinically significant anxiety; XGBoost, extreme gradient boosting; DT, decision tree; RF, random forest; SVM, support vector machine; KNN, k-nearest neighbor; AdaBoost, adaptive boosting; MLP, multilayer perceptron; NB naive Bayes; 7d, 7-day; wd, weekday; we, weekend.

The dataset was divided into a training set (*n* = 143, 69.8%) and a validation set (*n* = 62, 30.2%) using a random stratified sampling method. There were 23 participants with CSA in the training set and 10 in the validation set. In the validation set, as shown in Table 2 and Figure 4, the XGBoost model based on the record of accelerometer features on weekdays (XGBoost-wd) achieved the highest AUC value and F1 score, with values of 0.777 (95% CI, 0.591–0.963) and 0.546, respectively. The XGBoost models based on the record of accelerometer features on weekends (XGBoost-we), the 7-day period (XGBoost-7d), and the merged accelerometer features (XGBoost-merged) achieved AUC values and F1 scores of 0.567 (95% CI, 0.381–0.754) and 0.211, 0.715 (95% CI, 0.569–0.862) and 0.222, and 0.713 (95% CI, 0.537–0.890) and 0.435, respectively. Among the models based on the record of the 7-day period, XGBoost-7d performed the best. Additionally, among the models based on the record of accelerometer features on weekends, the AdaBoost model performed the best, with AUC values and F1 scores of 0.656 and 0.222, respectively. Among the models based on the merged accelerometer features, the DT model (DT-merged) performed the best, with an AUC value and F1 score of 0.735 and 0.483, respectively. The performance of the optimal models constructed using eight different ML algorithms is shown in Table 3 and Figure 4c,d. The performances of all the other models are shown in Supporting Information Table S3.

The DeLong test comparison results of the AUC values among all the models are shown in Supporting Information Figure S9. DCA revealed that at most risk thresholds, the XGBoost-wd model had the greatest net benefit compared with the XGBoost models constructed on other feature sets. When the risk threshold was below 35%, the DT-merged model had a net benefit comparable to that of the XGBoost model on the basis of weekday accelerometer features, but XGBoost-wd outperformed DT-merged when the risk threshold was in the range of 35%–50%.

**Figure 5:**
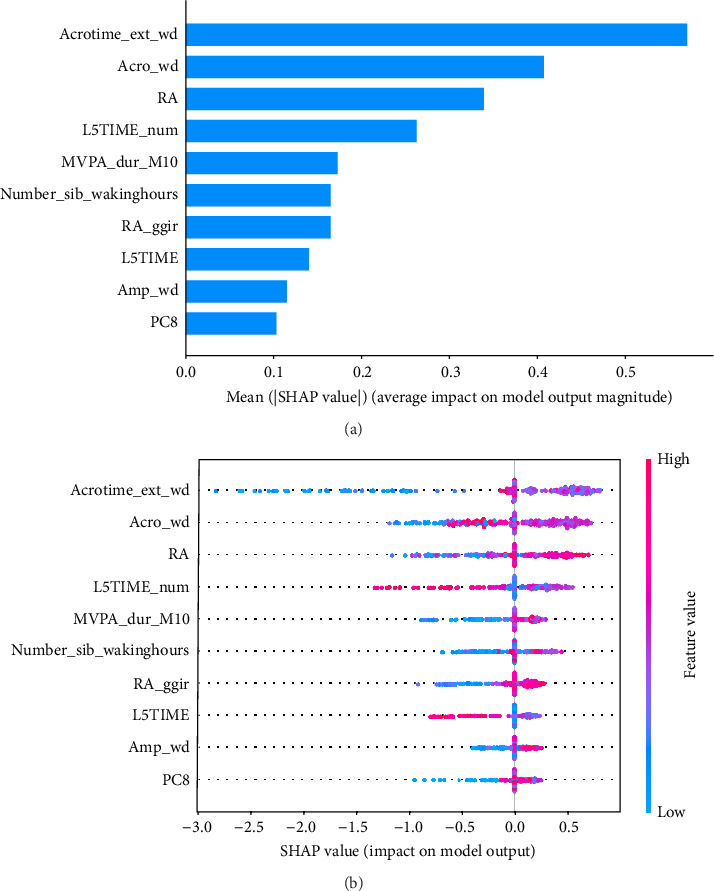
The contributions of the top 10 most important features in XGBoost-wd. (a) Global bar plot of the SHAP values from the XGBoost model. The features are arranged in descending order on the basis of their contributions from the model. (b) Beeswarm summary plot of SHAP values from the XGBoost model. The visualization illustrates the impact of these features on the model's predictions, with colors ranging from high values (red) to low values (blue). The horizontal position indicates whether the feature value contributes to a positive or negative prediction. Each point corresponds to the SHAP value of the feature for an individual instance. Acro: Acrophase, a measure of the time of the overall high values recurring in each cycle; RA, Relative amplitude; L5TIME_num, Timing of least active 5 h; MVPA_dur_M10, Total duration in minutes of moderate to vigorous activity within the M10 window; Number_sib_wakinghours, number of sustained inactivity bouts during the day, with day referring to the time outside the sleep period time window; amp, amplitude; wd, weekday; PC8, 8th principal component score from functional principal component analysis (FPCA).

As shown in Figure 5, SHAP analysis revealed that the five most important features in XGBoost-wd were Acrotime_ext_wd, Acro_wd, RA, L5TIME_num, and Number_sib_wakinghours. Among these features, Acrotime_ext_wd, Acro_wd, RA, and Number_sib_wakinghours were positively correlated with the occurrence of anxiety symptoms. Conversely, L5TIME_num was negatively correlated with CSA.

## 4. Discussion

There is a lack of understanding regarding the complex associations among CSA, SPs, PA, and CR, as well as the role of accelerometers in monitoring CSA in short-term insomnia. Thus, this cross-sectional study aimed to investigate the relationship between CSA and the subjective and objective parameters of sleep and the joint effect between CSA and accelerometer-measured features. Moreover, this study also established a series of predictive models with different periods of accelerometer-measured features using ML to identify CSA in patients with short-term insomnia.

### 4.1. Characteristics of the Subjective and Objective Sleep Parameters in Short-Term Insomnia Patients With CSA

In this study, short-term insomnia with CSA was associated with significantly worse subjective sleep problems. However, the TST was longer in those with an accelerometer than in those without anxiety. A possible explanation for this is that CSA may contribute to higher levels of both physical and psychological arousal before sleep [35], which could result in more time spent in bed trying to fall asleep, potentially delaying sleep onset [36, 37]. The accelerometer might mistakenly classify this time as sleep, thus leading to an overestimation of TST [38]. Furthermore, these behaviors could lead to severe insomnia severity and increase daytime sleepiness [39], reducing activity levels [40]. Another possible explanation for this is that CSA is linked to sleep state misperception [41], where individuals tend to underestimate their objective sleep duration [42, 43]. However, this study did not use self-reported sleep diaries to identify TST. Therefore, future studies should investigate the reasons for the discrepancies between subjective and objective sleep outcomes in short-term insomnia. The combination of a sleep diary and an accelerometer may be used to determine whether subjective sleep problems are not consistent with objective sleep.

This study revealed that short-term insomnia with CSA was associated with increased RA. RA describes the amplitude between the activity during the day and the night; a higher RA means an amplified CR amplitude, suggesting higher activity during the day and lower activity at night. We further compared the relative rest-activity features between short-term insomnia with and without CSA and found that anxiety symptoms were associated with increased average activity of the individual's most active 10 h and increased peak activity (amplitude). These findings are inconsistent with those of previous studies [6, 44, 45]. For example, Difrancesco et al. [6] reported that participants with depressive and/or anxiety disorders had lower RAs between day-time and night-time activities. Some studies also reported no significant associations between relative rest-activity features and anxiety [44, 45]. The variability could be due to the different populations used in these studies. Short-term insomnia can be seen as a physiological response to acute stressors or “threats” [46], which potentially lead to acute CR dysregulation [47, 48]. CSA in short-term insomnia may provoke stronger day‒night activity differences. However, anxiety disorders or CSA in individuals with chronic insomnia might lead to a more flattened CR [42, 49]. Further studies are needed to confirm the association between CR and CSA in short-term insomnia.

### 4.2. Interactive Effects of Accelerometer-Measured Features on CSA and Sleep Parameters

This study revealed that CSA and IS had an interactive association with sleep hygiene behaviors. For short-term insomnia patients with low IS, which means greater sleep‒wake pattern stability, anxiety symptoms were strongly associated with worse sleep behaviors. For those with high IS, no significant differences were found between anxiety symptoms and sleep behaviors. Unstable CR can lead to the dysregulation of neurotransmitters related to high CSA levels. For example, disruptions in serotonin levels can lead to CSA [50, 51], whereas fluctuations in dopamine can impair cognitive processes such as attention and memory [52, 53]. This dysregulation may result in dysfunctional cognition and maladaptive behaviors, as individuals struggle to manage their anxiety and cognition effectively. These anxiety-related beliefs and behaviors can further worsen sleep problems in individuals with short-term insomnia [53].

In addition, this study revealed that short-term insomnia participants with CSA presented longer sedentary time and lower MVPA time than those without CSA. Difrancesco et al. reported that anxiety disorders were associated with lower PAs [6 ], which aligns with the findings of this study [42, 54, 55]. Owing to maladaptive beliefs and attitudes, individuals with CSA may avoid stimulating or physically demanding activities, thinking that this will improve relaxation and help them fall asleep [56]. Our study also revealed that longer durations of PA, including both light and MVPA, had a stronger effect on the relationship between CSA and insomnia severity than did shorter durations of PA. Individuals with CSA tend to be more sensitive to interoception (the perception of the body's internal state, including gastric, respiratory and cardiac signals) [57]. A long PA duration may increase the heart rate or increase other physiological responses, which may further worsen interoception and lead to higher CSA levels among individuals with short-term insomnia. However, this study covered only a single dimension of PA, such as the duration of a specific activity. Thus, further studies are needed to identify and investigate activity profiles (e.g., frequency or record of exercise type) that have thus far been missed and to subsequently gain better insights into the role of PA behavior in short-term insomnia with CSA.

These findings indicate that there are tight and joint associations between anxiety symptoms in short-term insomnia and features such as SPs, PA, and CR. Therefore, it is necessary to predict CSA with integrated accelerometer information before treating short-term insomnia. Thus, this study constructed ML models on the basis of records of accelerometer features and observed whether records during different periods could positively influence the identification of CSA.

### 4.3. ML Models Using Accelerometer-Measured Features to Identify CSA in Short-Term Insomnia

We compared the classification performance of records of accelerometer features at different periods and various common ML algorithms in this task. To simplify the data processing procedure and increase the clinical utility, we directly use the outcomes processed using the R packages GGIR and postGGIR for model construction, including different periods of features on weekdays, weekends, and 7-day periods. Overall, in the ML models constructed in this study, most models exhibited better classification performance in identifying CSA when trained with weekday features. XGBoost-wd demonstrated the best classification performance and improved individuals' net benefits in the task of identifying CSA. For imbalanced data, accuracy was not a sufficient metric to fully evaluate the model. The model's F1 score reflected a good balance between correctly identifying true positives and minimizing false positives. This balance was essential for maintaining the model's reliability in practical applications, where both false positives and false negatives can have significant consequences.

The model results indicated that the record of accelerometer features has the potential to be used in assessing CSA, and the XGBoost models constructed with accelerometer features on weekdays had the best performance. A possible reason could be the discrepancy in the severity of anxiety symptoms and the accelerometer features between weekdays and weekends. Work-related activities on weekdays could increase the stress of short-term insomnia participants and negatively affect their mental and physical health [58]. Weekend activities could help recover energy and improve anxiety symptoms [59]. In clinical practice, this means that monitoring on weekdays may provide more accurate information about fluctuations in anxiety symptoms, which can help in developing personalized intervention plans. Clinicians can focus on weekday activity patterns to identify individuals at greater risk of anxiety and implement targeted interventions to reduce stress and improve mental health. Furthermore, long-term monitoring of accelerometer features is needed to explore the discrepancy between weekdays and weekends and help formulate individualized interventions [59, 60].

**Figure 6:**
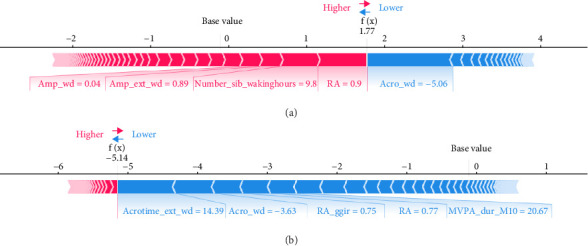
The force plot for the decision process of XGBoost-wd to identify whether two individuals in the validation set had CSA. Each feature provides an SHAP value to the model's baseline value. The final prediction value f(x) is obtained on the basis of the internal feature weights and calculations of the ML model. When f(x) >0, the model classifies the individual as having anxiety symptoms; otherwise, it classifies them as not having anxiety symptoms. (a) An individual with CSA. Owing to high RA, Number_sib_wakinghours, and Amp_ext_wd and a median value for Amp_wd, the model determines that the probability of this individual having anxiety symptoms is 85.4%. (b) An individual without CSA. Owing to low Acrotime_ext_wd, Acro_wd, RA_ggir, RA, and MVPA_dur_M10, the model determines that the probability of this individual not having anxiety symptoms is 99.4%. CSA, clinically significant anxiety.

ML models can provide users with the probability of anxiety risk for each individual, but this is not sufficient owing to a lack of interpretability. Therefore, in this study, we added the local explanation method SHAP to the ML models to show which variables and how they support the model in making decisions [16]. According to the SHAP value of XGBoost-wd, eight of the top ten features belong to the CR domain, suggesting the pivotal effects of circadian misalignment in predicting anxiety symptoms. Furthermore, PA and SP features also contribute to the risk of anxiety. Specifically, a later peak activity (acrophase), higher RAs and higher amplitude could increase the risk of anxiety. A shorter duration of MVPA and a later duration of the least active 5-h period could reduce the risk of anxiety. These results suggest that a more robust rest–activity pattern and nighttime rest period, reflecting both higher and earlier activity when awake and relatively lower activity during the night, are correlated with a decrease in the risk of anxiety symptoms.  Figure 6 displays the decision process of XGBoost-wd for two individuals in the validation set, visually illustrating the individualized risk factors that support the model's classification. Considering the complex joint associations among these features, those predicted to be at risk of anxiety using the XGBoost model may benefit from individualized interventions.

## 5. Limitations

Several limitations of this study require discussion. First, the cross-sectional design of this study limits our ability to investigate the temporal relationship between CSA and accelerometer-measured features. Therefore, future longitudinal studies are needed to track changes in accelerometer-measured features over time, which could help us clarify whether improvements in these features can reduce the risk of anxiety in individuals with CSA. Second, a 7-day wear period for the accelerometer should be considered for longer monitoring times to ensure more precise data collection. Third, this study did not use self-reported sleep diaries to investigate the discrepancies between subjective and objective sleep outcomes in short-term insomnia. Fourth, challenges remain in implementing accelerometer-based models in clinical practice, particularly the relatively high costs of accelerometers and the complexity of data analysis, both of which must be taken into account. Thus, further studies should apply larger samples and external validation to evaluate the generalizability of the constructed models [16 ]. Finally, although we combined accelerometer data with ML models, the clinical interpretability of certain features still requires further research, especially in real-world applications where clinicians may not be sufficiently familiar with ML methods.

## 6. Conclusion

This study confirmed that short-term insomnia with CSA significantly worsened subjective sleep problems, and accelerometer-measured features warrant further attention as interactive factors. Furthermore, this study suggests that accelerometer-measured features collected in daily life, combined with ML, potentially exhibit great clinical utility for accurately monitoring changes in sleep and identifying anxiety symptoms. In the future, more wearables and smartphones could embed related functions to collect abundant, varied patient accelerometer-measured features, capturing information more accurately, and conveniently than periodic clinical assessments. ML can process and analyze these collected data with complex relationships, providing clinicians with more easily understandable and usable explanation methods that enhance clinical decision-making.

## Figures and Tables

**Table 1:** Descriptive characteristics of adults with short-term insomnia.

Variable	Total (*n* = 205)	Without CSA group (*n* = 172)	CSA group (*n* = 33)	*p* Value
Age	29.00 (22.00−42.00)	28.00 (22.50−42.50)	29.00 (21.00−36.00)	0.273
BMI	21.48 (19.98−23.77)	21.48 (19.99−23.88)	21.08 (19.92−22.77)	0.328
Sex	—	—	—	0.027
Male	61 (29.76%)	57 (33.14%)	4 (12.12%)	—
Female	144 (70.24%)	115 (66.86%)	29 (87.88%)	—
Marriage status	—	—	—	1.000
Unmarried	82 (40.00%)	69 (40.12%)	13 (39.39%)	—
Married	113 (55.12%)	94 (54.65%)	19 (57.58%)	—
Divorce or others	10 (4.88%)	9 (5.23%)	1 (3.03%)	—
Education	—	—	—	0.318
College or above	187 (91.22%)	155 (90.12%)	32 (96.97%)	—
Senior high schools or below	18 (8.78%)	17 (9.88%)	1 (3.03%)	—
Job	—	—	—	0.544
Yes	105 (51.22%)	86 (50.00%)	19 (57.58%)	—
No	100 (48.78%)	86 (50.00%)	14 (42.42%)	—
ISI	15.00 (13.00−18.00)	14.00 (13.00−17.00)	18.00 (16.00; 21.00)	<0.001
FIRST	22.73 ± 5.27	22.01 ± 5.19	26.52 ± 3.98	<0.001
GSES	6.00 (4.00−8.00)	5.50 (4.00−8.00)	8.00 (6.00−9.00)	<0.001
DBAS	40.99±7.67	42.04±7.41	35.52±6.71	<0.001
MEQ	13.00 (12.00−15.00)	13.00 (12.00−15.00]	13.00 (12.00−15.00)	0.961
SHPS	32.60 ± 11.53	31.22 ± 11.00	39.82 ± 11.67	<0.001
PSAS	34.65 ± 7.82	47.42 ± 8.10	36.70 ± 9.15	<0.001
ESS	8.42 ± 4.11	8.13 ± 4.00	9.94 ± 4.40	0.020
PHQ	8.00 (5.00−10.00)	7.00 (5.00−9.00)	13.00 (10.00−16.00)	<0.001
GAD	5.00 (2.00−8.00)	4.00 (2.00−7.00)	13.00 (11.00−15.00)	<0.001
TST	6.37 (5.82; 6.84)	6.34 (5.71−6.83)	6.53 (6.10−7.13)	0.047
SE	0.85 (0.81−0.89)	0.86 (0.80−0.89)	0.85 (0.81−0.89)	0.648
Sedentary time	789.52 ± 105.94	752.26 ± 124.06	789.52 ± 105.94	0.027
Light activity time	230.62 (188.38−276.38)	224.62 (188.38−281.88)	230.62 (188.38−276.38)	0.873
MVPA time	65.50 (46.00−87.38)	75.50 (58.12−107.57)	65.50 (46.00−87.38)	0.004
RA	0.80 (0.76−0.83)	0.79 (0.75−0.83)	0.84 (0.80−0.86)	<0.001
IV	0.81±0.16	0.81±0.16	0.79±0.16	0.427
IS	0.35 (0.30−0.41)	0.35 (0.30−0.41)	0.36 (0.32−0.42)	0.315
Accelerometer valid time hours/per day	22.98 ± 2.57	23.00 ± 2.26	22.91 ± 3.87	0.864

Abbreviations: BMI, Body Mass Index; CSA, clinically significant anxiety; DBAS, dysfunctional beliefs and attitudes; ESS, Epworth Sleepiness Scale; FIRST, Ford Insomnia Response to Stress Test; GAD-7, 7-item Generalized Anxiety Disorder Questionnaire; GSES, Glasgow Sleep Effort Scale; IS, interdaily stability; ISI, Insomnia Severity Index; IV, intradaily variability; MEQ, Morningness–Eveningness Questionnaire; MVPA, moderate to vigor activity; PHQ-9, 9-item Patient Health Questionnaire; PSAS, presleep arousal scale; RA, Relative amplitude; SE, sleep efficiency; SHPS, Sleep Hygiene Practice Scale; TST, total sleep time.

**Table 2:** Classification performance of the four feature sets in identifying CSA.

	AUC	F1_Score	Youden	Sensitivity	Specificity	Accuracy
XGBoost-7d	0.713 (0.537, 0.890)	0.435	0.346	0.500 (0.187, 0.813)	0.846 (0.719, 0.931)	0.790 (0.668, 0.883)
XGBoost-wd	0.777 (0.591, 0.963)	0.545	0.485	0.600 (0.262, 0.878)	0.885 (0.766, 0.956)	0.839 (0.723, 0.920)
XGBoost-we	0.567 (0.381, 0.754)	0.211	0.065	0.200 (0.025, 0.556)	0.865 (0.742, 0.944)	0.758 (0.633, 0.858)
XGBoost-merged	0.715 (0.569, 0.862)	0.222	0.085	0.200 (0.025, 0.556)	0.885 (0.766, 0.956)	0.774 (0.650, 0.871)
DT-merged	0.735 (0.574, 0.895)	0.483	0.469	0.700 (0.348, 0.933)	0.769 (0.632, 0.875)	0.758 (0.633, 0.858)
AdaBoost-we	0.656 (0.476, 0.835)	0.222	0.085	0.200 (0.025, 0.556)	0.885 (0.766, 0.956)	0.774 (0.650, 0.871)

Abbreviations: 7d: 7-day; AdaBoost: adaptive boosting; AUC: area under curve; DT: decision tree; wd: weekday; we: weekend; XGBoost: extreme gradient boosting.

**Table 3:** Classification performance of 8 ML algorithms in identifying CSA.

	AUC	F1_Score	Youden	Sensitivity	Specificity	Accuracy
XGBoost-wd	0.777 (0.591, 0.963)	0.545	0.485	0.600 (0.262, 0.878)	0.885 (0.766, 0.956)	0.839 (0.723, 0.920)
DT-merged	0.735 (0.574, 0.895)	0.483	0.469	0.700 (0.348, 0.933)	0.769 (0.632, 0.875)	0.758 (0.633, 0.858)
RF-wd	0.712 (0.532, 0.891)	0.417	0.327	0.500 (0.187, 0.813)	0.827 (0.697, 0.918)	0.774 (0.650, 0.871)
SVM-wd	0.631 (0.413, 0.849)	0.345	0.231	0.500 (0.187, 0.813)	0.731 (0.590, 0.844)	0.694 (0.563, 0.804)
KNN-wd	0.726 (0.557, 0.895)	0.410	0.396	0.800 (0.444, 0.975)	0.596 (0.451, 0.730)	0.629 (0.497, 0.748)
AdaBoost-wd	0.715 (0.538, 0.893)	0.417	0.327	0.500 (0.187, 0.813)	0.827 (0.697, 0.918)	0.774 (0.650, 0.871)
MLP-wd	0.769 (0.629, 0.909)	0.385	0.288	0.500 (0.187, 0.813)	0.788 (0.653, 0.889)	0.742 (0.615, 0.845)
NB-7d	0.672 (0.464, 0.880)	0.414	0.350	0.600 (0.262, 0.878)	0.750 (0.611, 0.860)	0.726 (0.598, 0.831)

Abbreviations: 7d: 7-day; AdaBoost: adaptive boosting; AUC: area under the curve; DT: decision tree; KNN: *k*-nearest neighbor; MLP: multilayer perceptron; NB: naive Bayes; RF: random forest; SVM: support vector machine; wd: weekday; we: weekend; XGBoost: extreme gradient boosting.

## Data Availability

The data that support the findings of this study are available from the corresponding author upon reasonable request.

## Supporting Information

Supporting Information Supporting Information S1. Concise explanation of each metric of the models. Supporting Information Figure S1: Sample size estimation for the mRS prediction model based on MedCalc. Supporting Information Figure S2: shows the forest plot estimating the FIRST scores according to joint associations of accelerometer-measured features and CSA. Supporting Information Figure S3: shows the forest plot estimating the GSES scores according to joint associations of accelerometer-measured features and CSA. Supporting Information Figure S4: shows the forest plot estimating the DBAS scores according to joint associations of accelerometer-measured features and CSA. Supporting Information Figure S5: shows the forest plot estimating the PSAS scores according to joint associations of accelerometer-measured features and CSA. Supporting Information Figure S6: shows the forest plot estimating the MEQ scores according to joint associations of accelerometer-measured features and CSA. Supporting Information Figure S7: shows the forest plot estimating the ESS scores according to joint associations of accelerometer-measured features and CSA. Supporting Information Figure S8: shows the forest plot estimating the TST according to joint associations of accelerometer-measured features and CSA. Supporting Information Figure S9: shows the forest plot estimating the SE according to joint associations of accelerometer-measured features and CSA. Supporting Information Figure S10: shows the heatmap of the Delong test results for the AUC between all the models. Supporting Information Table S1: shows a description of the accelerometer-measured features. Supporting Information Table S2: shows the missing data for the CSA classification tasks. Supporting Information Table S3: shows the classification performance for diagnosing CSA.
