# Supplementary material for: Machine Learning Models to Identify Clinically Significant Anxiety in Short-Term Insomnia Using Accelerometers
Source: Depress Anxiety. 2025 May 13;2025:3082856. doi: 10.1155/da/3082856 (PMC12092156; doi:10.1155/da/3082856)
Supplement: Supporting Information — S1. Concise explanation of each metric of the models. Supporting Information Figure S1: Sample size estimation for the mRS prediction model based on MedCalc. Supporting Information Figure S2: shows the forest plot estimating the FIRST scores according to joint associations of accelerometer-measured features and CSA. Supporting Information Figure S3: shows the forest plot estimating the GSES scores according to joint associations of accelerometer-measured features and CSA. Supporting Information Figure S4: shows the forest plot estimating the DBAS scores according to joint associations of accelerometer-measured features and CSA. Supporting Information Figure S5: shows the forest plot estimating the PSAS scores according to joint associations of accelerometer-measured features and CSA. Supporting Information Figure S6: shows the forest plot estimating the MEQ scores according to joint associations of accelerometer-measured features and CSA. Supporting Information Figure S7: shows the forest plot estimating the ESS scores according to joint associations of accelerometer-measured features and CSA. Supporting Information Figure S8: shows the forest plot estimating the TST according to joint associations of accelerometer-measured features and CSA. Supporting Information Figure S9: shows the forest plot estimating the SE according to joint associations of accelerometer-measured features and CSA. Supporting Information Figure S10: shows the heatmap of the Delong test results for the AUC between all the models. Supporting Information Table S1: shows a description of the accelerometer-measured features. Supporting Information Table S2: shows the missing data for the CSA classification tasks. Supporting Information Table S3: shows the classification performance for diagnosing CSA. [file 3082856.f1.docx]

**Supplementary Material 1**

Below is a concise explanation of each metric:

- **AUC (Area Under the ROC Curve)**: Quantifies the overall performance of a binary classifier across all possible thresholds. A higher AUC indicates better disc**riminative ability.**
- **Decision Curve Analysis (DCA): Evaluates the net clinical benefit of a predictive model at various probability thresholds, helping assess its practical utility.**
- **F1 Score: The harmonic mean of precision and sensitivity**

$$(2\times\frac{Precision\times Ssensitivity}{Precision+Ssensitivity})$$

- **Youden Index: Reflecting a test’s overall discriminative power, calculated as**

$$(Sensitivity+Specificity-1)$$

- **Sensitivity: The proportion of actual positives that are correctly identified as such**

$$(\frac{TP}{TP+FN})$$

- **Specificity: The proportion of actual negatives that are correctly identified as such**

$$(\frac{TN}{TN+FP})$$

- **Accuracy: The overall fraction of correct predictions that are correctly identified as such**

$$(\frac{TP+TN}{TP+TN+FP+FN})$$

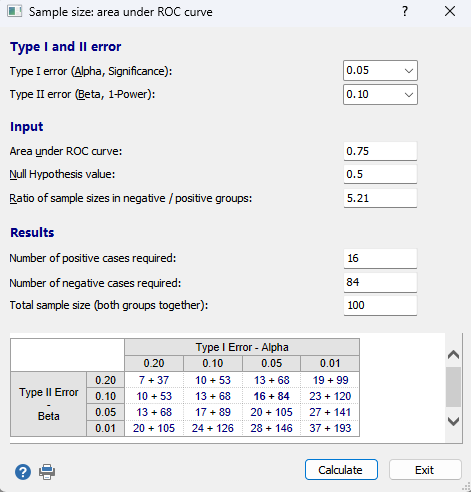


**Supplementary Figure 1.** Sample size estimation for the mRS prediction model based on MedCalc.


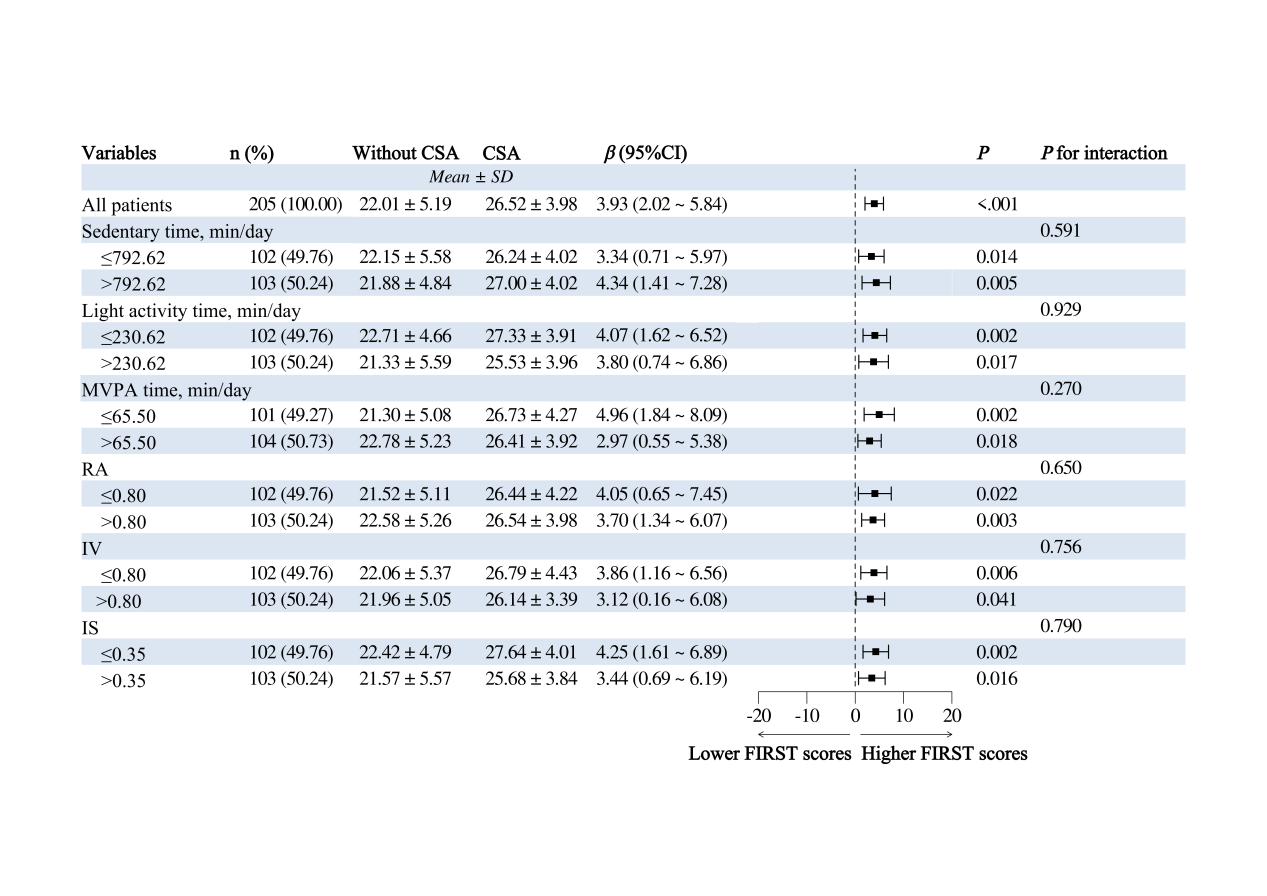


**Supplementary Figure 2.** Forest plot estimating the FIRST scores according to joint associations of accelerometer-measured features and CSA. β and 95% confidence intervals were weighted to be nationally representative. Linear regression models were adjusted for age, sex, education, BMI, marriage, education and job. MVPA time defined as low(≤65.50 min/day) or high (>65.50 min/day) according to sample median. Sedentary time defined as low (≤792.62 min/day) or high (>792.62 min/day) according to sample median. RA defined as low(≤0.80) and high (>0.80) according to sample median. IV defined as low(≤0.80) and high (>0.80) according to sample median. IS defined as low(≤0.35) and high (>0.35) according to sample median; FIRST: Ford insomnia response to stress test; CSA: clinically significant anxiety;MVPA: moderate to vigorous physical activity; RA: Relative amplitude; IV: Intra-daily variability; IS: Inter-daily stability;MVPA: moderate to vigorous physical activity; RA: Relative amplitude; IV: Intradaily variability; IS: Interdaily stability.


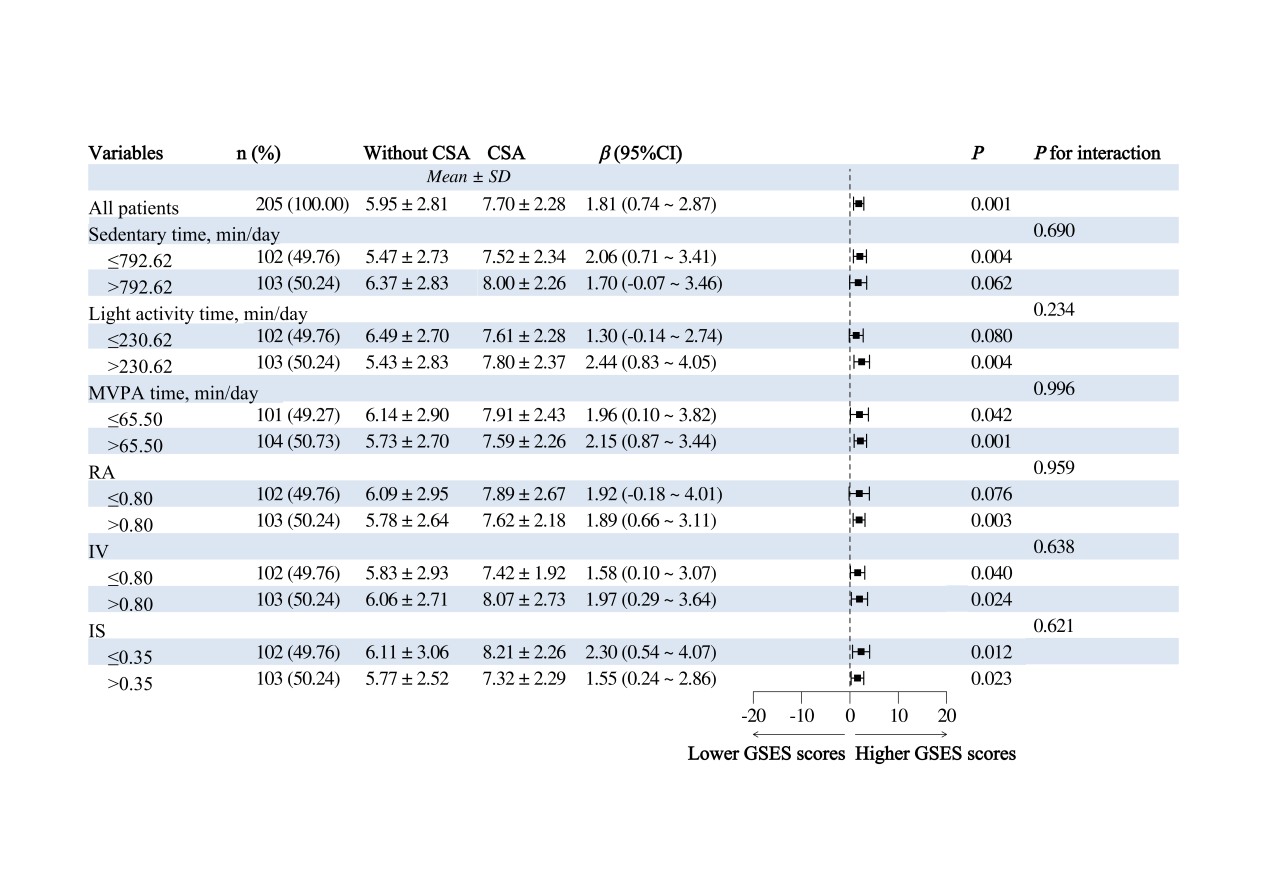


**Supplementary Figure 3.** Forest plot estimating the GSES scores according to joint associations of accelerometer-measured features and CSA. β and 95% confidence intervals were weighted to be nationally representative. Linear regression models were adjusted for age, sex, education, BMI, marriage, education and job. MVPA time defined as low(≤65.50 min/day) or high (>65.50 min/day) according to sample median. Sedentary time defined as low (≤792.62 min/day) or high (>792.62 min/day) according to sample median. RA defined as low(≤0.80) and high (>0.80) according to sample median. IV defined as low(≤0.80) and high (>0.80) according to sample median. IS defined as low(≤0.35) and high (>0.35) according to sample median; GSES: Glasgow Sleep Effort Scale;CSA: clinically significant anxiety;MVPA: moderate to vigorous physical activity; RA: Relative amplitude; IV: Intradaily variability; IS: Interdaily stability.


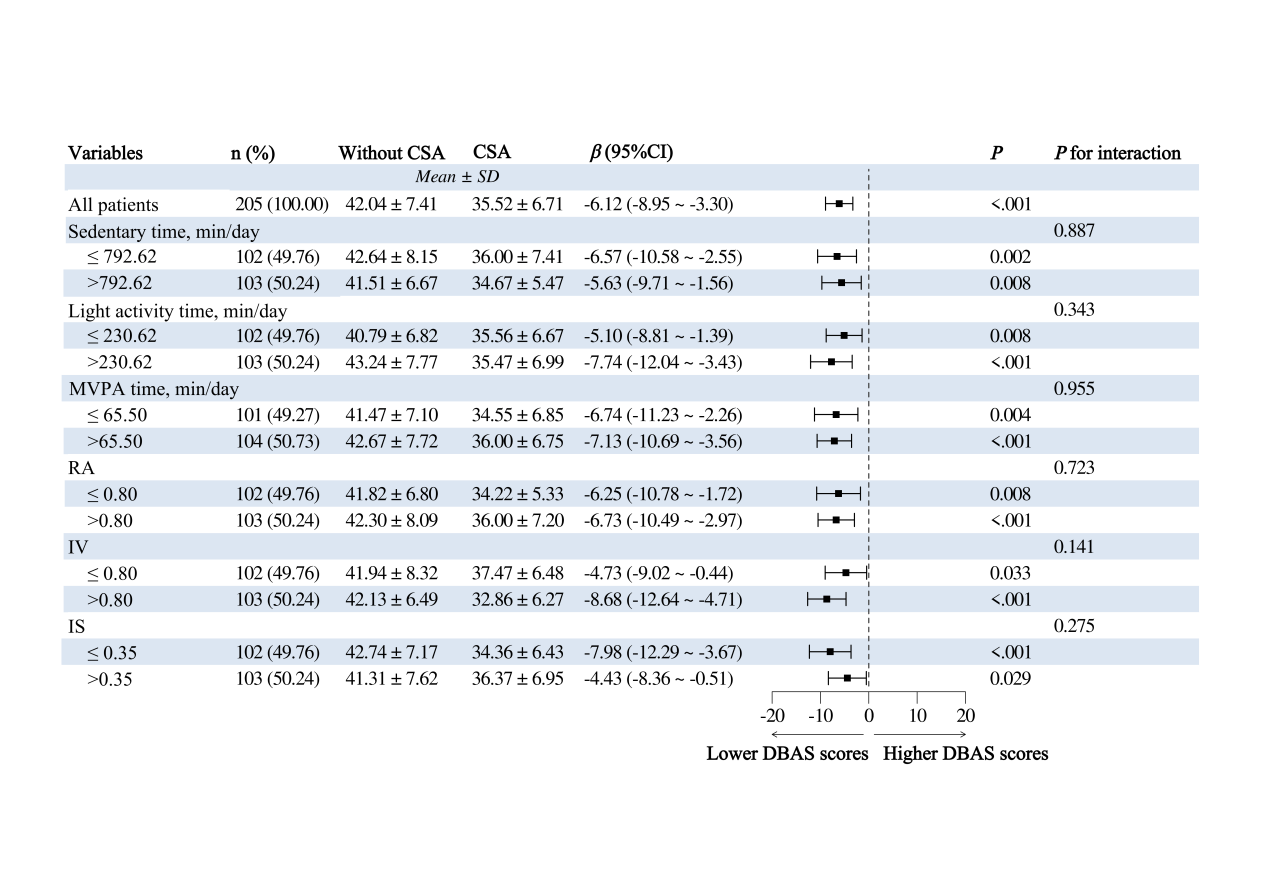


**Supplementary Figure 4.** Forest plot estimating the DBAS scores according to joint associations of accelerometer-measured features and CSA. β and 95% confidence intervals were weighted to be nationally representative. Linear regression models were adjusted for age, sex, education, BMI, marriage, education and job. MVPA time defined as low(≤65.50 min/day) or high (>65.50 min/day) according to sample median. Sedentary time defined as low (≤792.62 min/day) or high (>792.62 min/day) according to sample median. RA defined as low(≤0.80) and high (>0.80) according to sample median. IV defined as low(≤0.80) and high (>0.80) according to sample median. IS defined as low(≤0.35) and high (>0.35) according to sample median; DBAS: Dysfunctional beliefs and attitudes; CSA: clinically significant anxiety;MVPA: moderate to vigorous physical activity; RA: Relative amplitude; IV: Intradaily variability; IS: Interdaily stability.


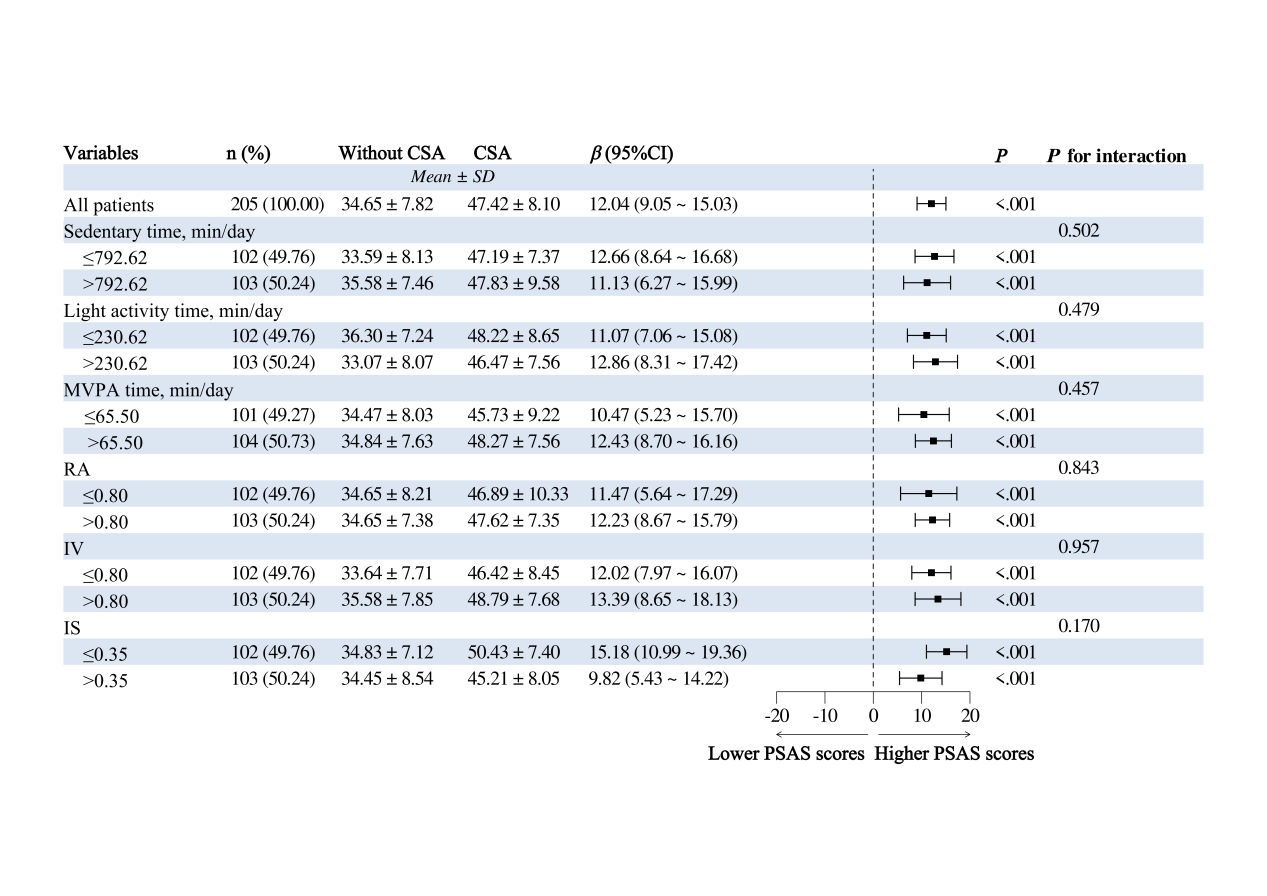


**Supplementary Figure 5.** Forest plot estimating the PSAS scores according to joint associations of accelerometer-measured features and CSA. β and 95% confidence intervals were weighted to be nationally representative. Linear regression models were adjusted for age, sex, education, BMI, marriage, education and job. MVPA time defined as low(≤65.50 min/day) or high (>65.50 min/day) according to sample median. Sedentary time defined as low (≤792.62 min/day) or high (>792.62 min/day) according to sample median. RA defined as low(≤0.80) and high (>0.80) according to sample median. IV defined as low(≤0.80) and high (>0.80) according to sample median. IS defined as low(≤0.35) and high (>0.35) according to sample median.; PSAS: pre-sleep arousal scale; CSA: clinically significant anxiety;MVPA: moderate to vigorous physical activity; RA: Relative amplitude; IV: Intradaily variability; IS: Interdaily stability.


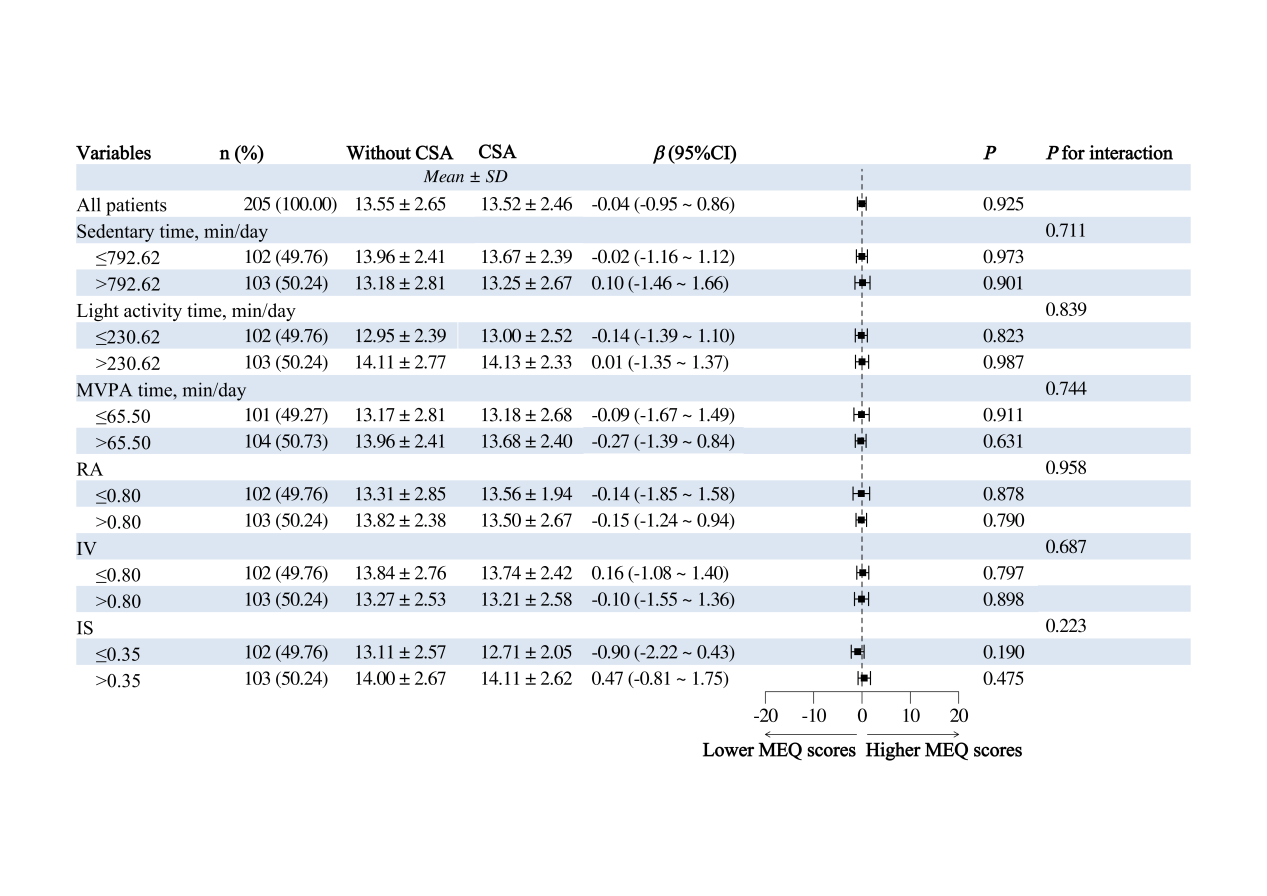


**Supplementary Figure 6.** Forest plot estimating the MEQ scores according to joint associations of accelerometer-measured features and CSA. β and 95% confidence intervals were weighted to be nationally representative. Linear regression models were adjusted for age, sex, education, BMI, marriage, education and job. MVPA time defined as low(≤65.50 min/day) or high (>65.50 min/day) according to sample median. Sedentary time defined as low (≤792.62 min/day) or high (>792.62 min/day) according to sample median. RA defined as low(≤0.80) and high (>0.80) according to sample median. IV defined as low(≤0.80) and high (>0.80) according to sample median. IS defined as low(≤0.35) and high (>0.35) according to sample median; MEQ: Morningness-Eveningness Questionnaire; CSA: clinically significant anxiety;MVPA: moderate to vigorous physical activity; RA: Relative amplitude; IV: Intradaily variability; IS: Interdaily stability.


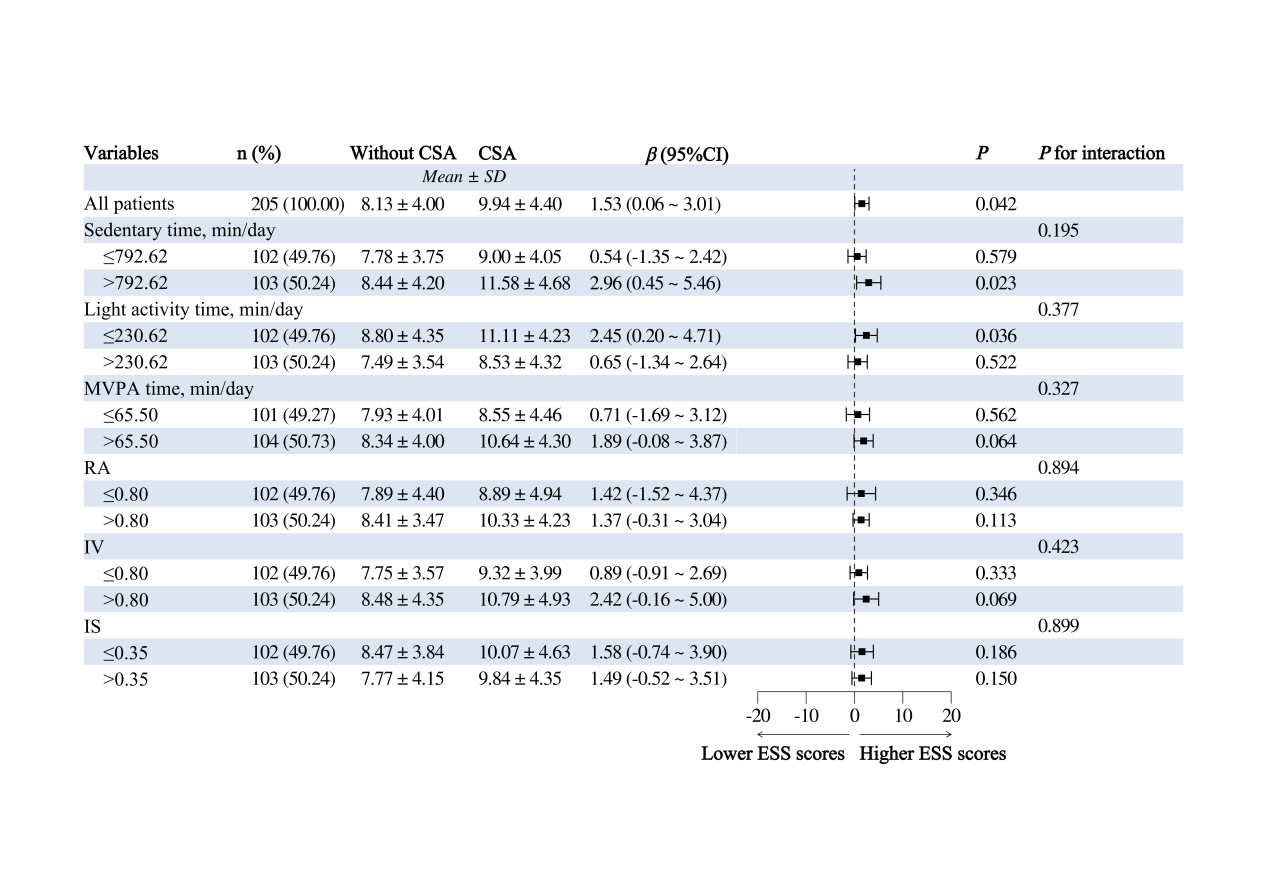


**Supplementary Figure 7.** Forest plot estimating the ESS scores according to joint associations of accelerometer-measured features and CSA. β and 95% confidence intervals were weighted to be nationally representative. Linear regression models were adjusted for age, sex, education, BMI, marriage, education and job. MVPA time defined as low(≤65.50 min/day) or high (>65.50 min/day) according to sample median. Sedentary time defined as low (≤792.62 min/day) or high (>792.62 min/day) according to sample median. RA defined as low(≤0.80) and high (>0.80) according to sample median. IV defined as low(≤0.80) and high (>0.80) according to sample median. IS defined as low(≤0.35) and high (>0.35) according to sample median.;ESS: Epworth Sleepiness Scale; CSA: clinically significant anxiety;MVPA: moderate to vigorous physical activity; RA: Relative amplitude; IV: Intradaily variability; IS: Interdaily stability.


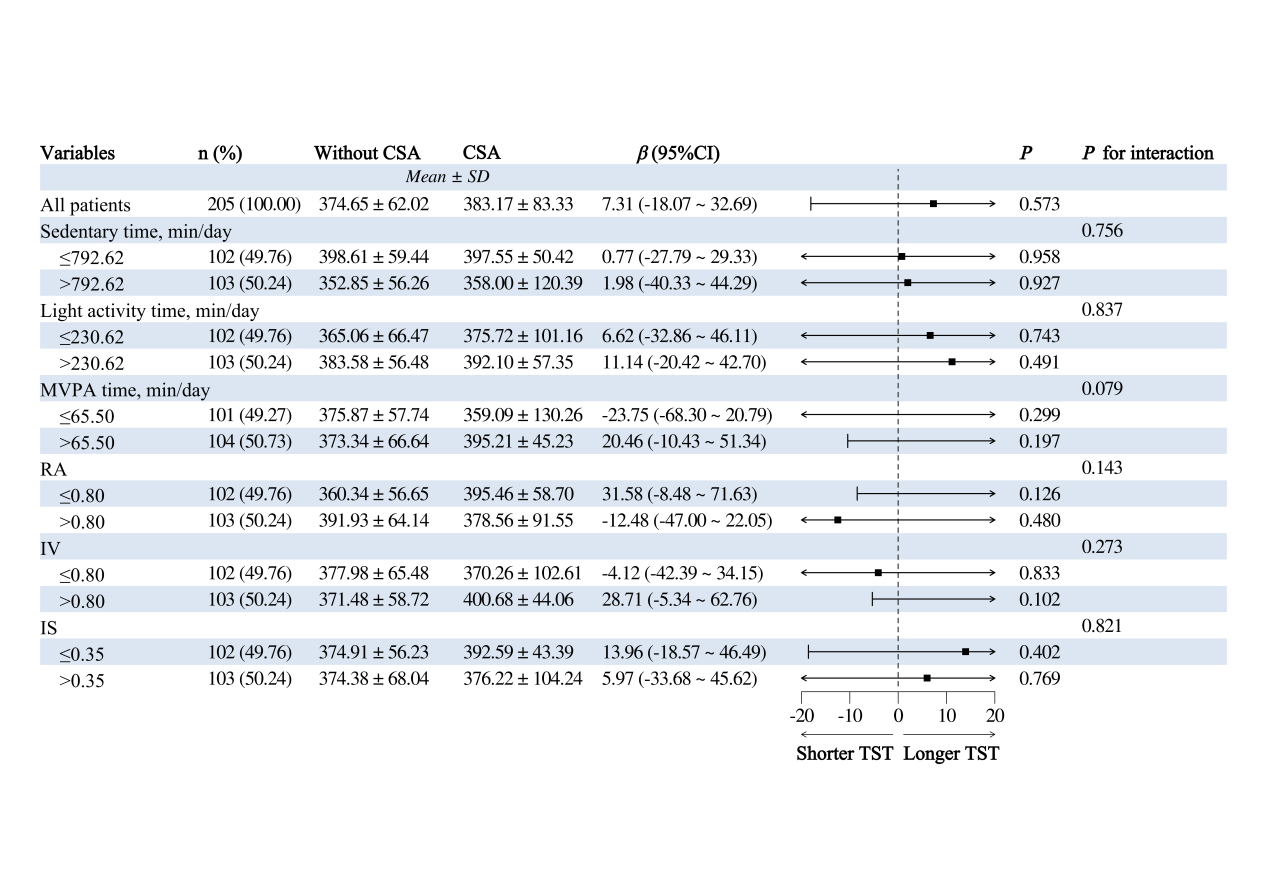


**Supplementary Figure 8.** Forest plot estimating the TST according to joint associations of accelerometer-measured features and CSA. β and 95% confidence intervals were weighted to be nationally representative. Linear regression models were adjusted for age, sex, education, BMI, marriage, education and job. TST, total sleep time per day estimated by the accelerometer. MVPA time defined as low(≤65.50 min/day) or high (>65.50 min/day) according to sample median. Sedentary time defined as low (≤792.62 min/day) or high (>792.62 min/day) according to sample median. RA defined as low(≤0.80) and high (>0.80) according to sample median. IV defined as low(≤0.80) and high (>0.80) according to sample median. IS defined as low(≤0.35) and high (>0.35) according to sample median.;TST: total sleep time; CSA: clinically significant anxiety;MVPA: moderate to vigorous physical activity; RA: Relative amplitude; IV: Intradaily variability; IS: Interdaily stability.


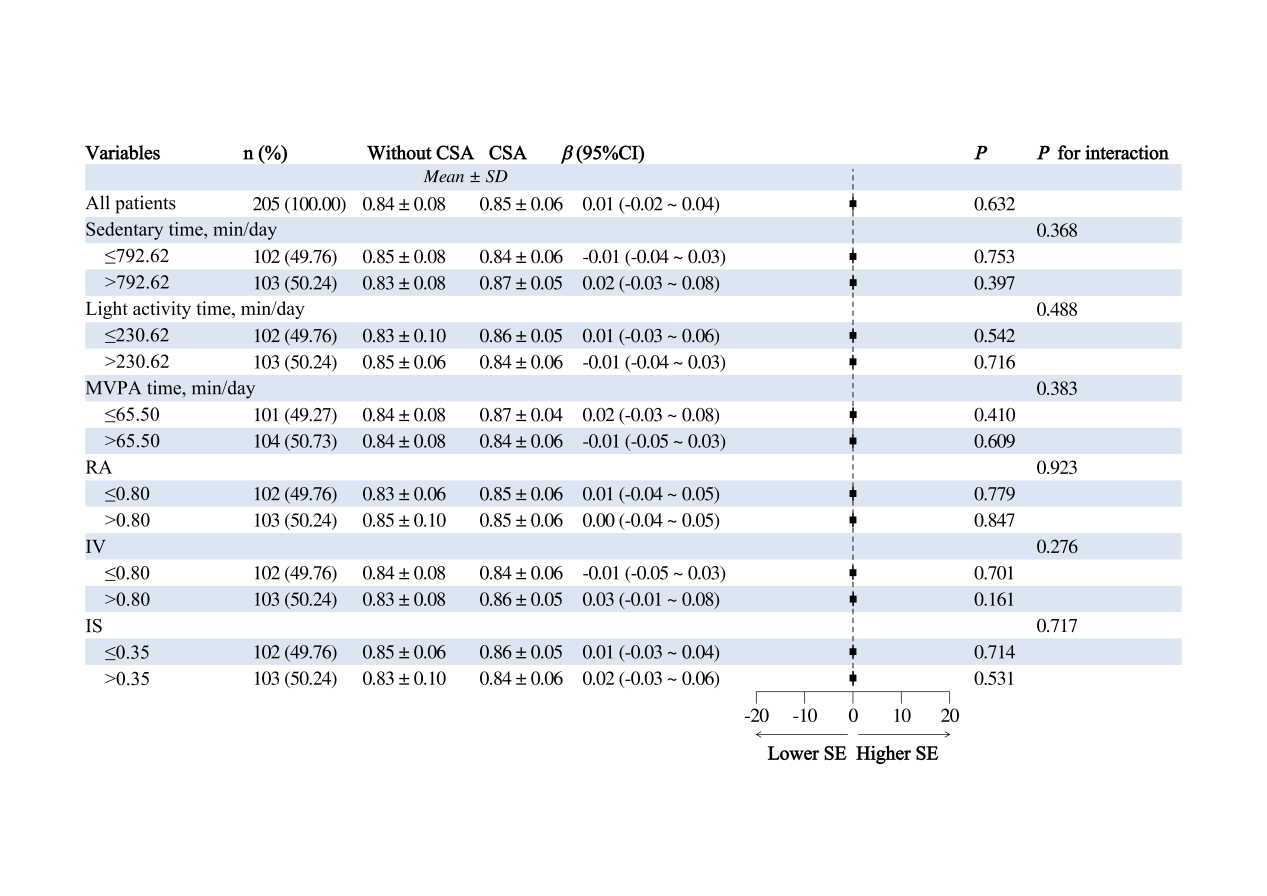


**Supplementary Figure 9.** Forest plot estimating the SE according to joint associations of accelerometer-measured features and CSA. β and 95% confidence intervals were weighted to be nationally representative. Linear regression models were adjusted for age, sex, education, BMI, marriage, education and job. SE, sleep efficiency per day estimated by the accelerometer. MVPA time defined as low(≤65.50 min/day) or high (>65.50 min/day) according to sample median. Sedentary time defined as low (≤792.62 min/day) or high (>792.62 min/day) according to sample median. RA defined as low(≤0.80) and high (>0.80) according to sample median. IV defined as low(≤0.80) and high (>0.80) according to sample median. IS defined as low(≤0.35) and high (>0.35) according to sample median.;SE: sleep efficiency; CSA: clinically significant anxiety;MVPA: moderate to vigorous physical activity; RA: Relative amplitude; IV: Intradaily variability; IS: Interdaily stability.


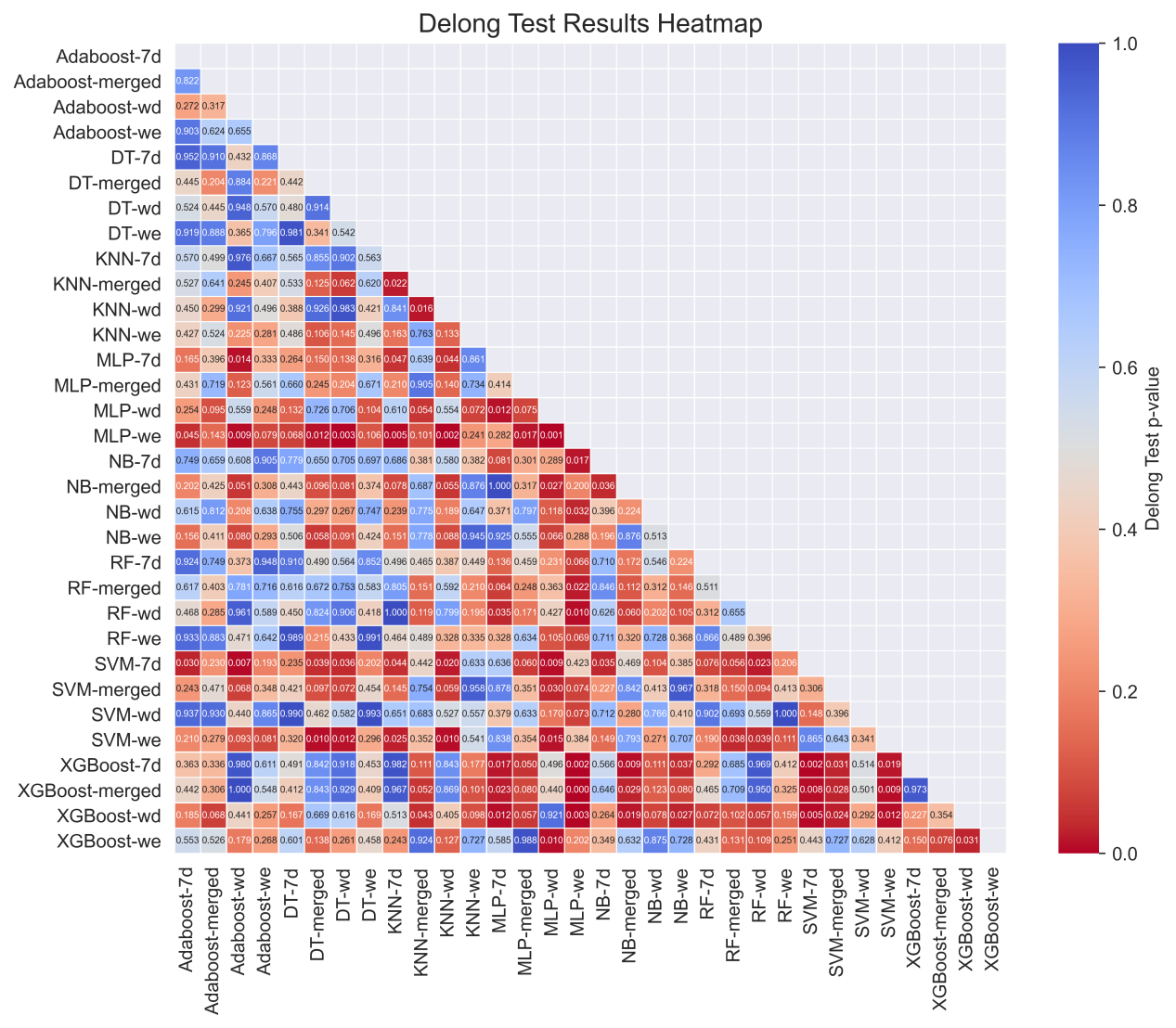


**Supplementary Figure 10. The heatmap of Delong test results for AUC between all models.**

Abbrevation: XGBoost: extreme gradient boosting; DT: decision tree; RF: random forest; SVM: support vector machine; KNN: *k*-nearest neighbor; Adaboost: adaptive boosting; MLP: multilayer perceptron; NB: naive bayes; wd: weekday; we: weekend..

**Supplementary Table 1.** The description of accelerometer-measured features.

| **Domain** | **Variable** | **Description** |
| --- | --- | --- |
| Sleep | sleeponset | Detected onset of sleep expressed as hours since the midnight of the previous night. |
|  | wakeup | Detected waking time (after sleep period) expressed as hours since the midnight of the previous night. |
|  | number_sib_wakinghours | Number of sustained inactivity bouts during the day, with day referring to the time outside the Sleep Period Time window. |
|  | TST | Total sleep time, which equals the accumulated nocturnal sustained inactivity bouts within the Sleep Period Time |
|  | sleep_Midpoint | Sleep Midpoint = (sleeponset + wakeup)/2. |
|  | SE | Sleep Efficiency = TST / (wakeup - sleeponset), which was the fraction of time spent asleep in the Sleep Period Time window. |
|  | N_atleast5minwakenight | Number of times awake during the night for at least 5 minutes |
|  | ACC_spt_sleep_mg | Average acceleration during sleep (mg) |
|  | Nblocks_spt_sleep | Number of blocks of night sleep within the Sleep period time window. |
| Physical Activity | TAC | Total volume of physical activity in the daytime |
|  | TLAC | Total volume of log-transformed physical activity in the daytime |
|  | TAC_24h | Total volume of physical activity within 24 hours |
|  | TLAC_24h | Total volume of log-transformed physical activity within 24 hours |
|  | Sedentary time | Total daytime duration in minutes of sedentary activity |
|  | Light activity time | Total daytime duration in minutes of light activity |
|  | mod_dur | Total daytime duration in minutes of moderate activity |
|  | vig_dur | Total daytime duration in minutes of vigorous activity |
|  | MVPA time | Total daytime duration in minutes of moderate to vigorous activity |
|  | sed_dur_M10 | Total duration in minutes of sedentary activity within M10 window |
|  | light_dur_M10 | Total duration in minutes of light activity within M10 window |
|  | mod_dur_M10 | Total duration in minutes of moderate activity within M10 window |
|  | vig_dur_M10 | Total duration in minutes of vigorous activity within M10 window |
|  | MVPA_dur_M10 | Total duration in minutes of moderate to vigorous activity within M10 window |
|  | sed_dur_24h | Total duration in minutes of sedentary activity within 24 hours |
|  | light_dur_24h | Total duration in minutes of light activity within 24 hours |
|  | mod_dur_24h | Total duration in minutes of moderate activity within 24 hours |
|  | vig_dur_24h | Total duration in minutes of vigorous activity within 24 hours |
|  | MVPA_dur_24h | Total duration in minutes of moderate to vigorous activity within 24 hours |
|  | mean_r | Mean sedentary bout duration. |
|  | mean_a | Mean active bout duration. |
|  | SATP | Sedentary to active transition probabilities. |
|  | ASTP | Active to sedentary transition probabilities. |
|  | Gini_r | Gini index for active bout, absolute variability normalized to the average bout duration (resting). |
|  | Gini_a | Gini index for sedentary bout, absolute variability normalized to the average bout duration (active). |
|  | alpha_r | Power law parameter for sedentary bout. |
|  | alpha_a | Power law parameter for active bout. |
|  | h_r | Hazard function for sedentary bout. |
|  | h_a | Hazard function for active bout. |
|  | dur_day_total_IN_min | Total duration of day in minutes spent in total inactivity during the day |
|  | dur_day_total_LIG_min | Total duration of day in minutes of light activity during the day |
|  | dur_day_total_MOD_min | Total duration of day in minutes of moderate activity during the day |
|  | dur_day_total_VIG_min | Total duration of day in minutes of vigorous activity during the day |
|  | dur_day_MVPA_bts_10_min | Total duration in minutes of Moderate and Vigorous Physical Activity (MVPA) for bouts 10 minutes or more |
|  | dur_day_MVPA_bts_5_10_min | Total duration in minutes of Moderate and Vigorous Physical Activity (MVPA) for bouts 5 to 10 minutes |
|  | dur_day_MVPA_bts_1_5_min | Total duration in minutes of Moderate and Vigorous Physical Activity (MVPA) for bouts 1 to 5 minutes |
|  | Nbouts_day_IN_bts_30 | Number of bouts of inactivity for bouts 30 minutes or more |
|  | Nbouts_day_IN_bts_20_30 | Number of bouts of inactivity for bouts 20 to 30 minutes |
|  | Nbouts_day_IN_bts_10_20 | Number of bouts of inactivity for bouts 10 to 20 minutes |
|  | Nbouts_day_LIG_bts_10 | Number of bouts of light for bouts 10 minutes or more |
|  | Nbouts_day_LIG_bts_5_10 | Number of bouts of light for bouts 5 to 10 minutes |
|  | Nbouts_day_LIG_bts_1_5 | Number of bouts of light for bouts 1 to 5 minutes |
|  | Nbouts_day_MVPA_bts_10 | Number of bouts of Moderate and Vigorous Physical Activity (MVPA) for bouts 10 minutes or more |
|  | Nbouts_day_MVPA_bts_5_10 | Number of bouts of Moderate and Vigorous Physical Activity (MVPA) for bouts 5 to 10 minutes |
|  | Nbouts_day_MVPA_bts_1_5 | Number of bouts of Moderate and Vigorous Physical Activity (MVPA) for bouts 1 to 5 minutes |
|  | Nblocks_day_total_IN | Number of blocks of total inactivity during day |
|  | Nblocks_day_total_LIG | Number of blocks of total light activity during day |
|  | Nblocks_day_total_MOD | Number of blocks of total moderate activity during day |
|  | Nblocks_day_total_VIG | Number of blocks of total vigorous activity during day |
| Circadian Rhythmicity | L5TIME_num | Timing of least active 5 hours |
|  | M10TIME_num | Timing of most active 10 hours |
|  | L5VALUE | Average acceleration value (mg) of least active 5 hours |
|  | M10VALUE | Average acceleration value (mg) of most active 10 hours |
|  | RA_ggir | Relative amplitude reflects the difference between M10 activity and L5 activity and RA_ggir = (M10VALUE-L5VALUE)/(M10VALUE+L5VALUE). |
|  | L5 | Average acceleration value (mg) of least active 5 hours |
|  | M10 | Average acceleration value (mg) of most active 10 hours |
|  | L5TIME | Timing of least active 5 hours |
|  | M10TIME | Timing of most active 10 hours |
|  | RA | Relative amplitude: (M10-L5)/(M10+L5) where M10= most active 10 hrs, L5 = least active 5 hour. |
|  | IV | Intra-daily variability measures fragmentation in the rest/activity rhythms. |
|  | IS | Inter-daily stability measures fragmentation in the rest/activity rhythms between different days. |
|  | mesor | MESOR which is short for midline statistics of rhythm, which is a rhythm adjusted mean. This represents mean activity level. |
|  | amp | Amplitude, a measure of half the extend of predictable variation within a cycle. This represents the highest activity one can achieve. |
|  | acro | Acrophase, a measure of the time of the overall high values recurring in each cycle. Here it has a unit of radian. This represents time to reach the peak. |
|  | mesor_L | Mesor in the cosinor model by using log-transformed data. |
|  | amp_L | Amplitude log-transformed in the cosinor model by using log-transformed data. |
|  | acro_L | Acrophase log-transformed in the cosinor model by using log-transformed data. |
|  | PC1 | 1st principal component score from functional principal component analysis (FPCA). |
|  | PC2 | 2nd principal component score from functional principal component analysis (FPCA). |
|  | PC3 | 3rd principal component score from functional principal component analysis (FPCA). |
|  | PC4 | 4th principal component score from functional principal component analysis (FPCA). |
|  | PC5 | 5th principal component score from functional principal component analysis (FPCA). |
|  | PC6 | 6th principal component score from functional principal component analysis (FPCA). |
|  | PC7 | 7th principal component score from functional principal component analysis (FPCA). |
|  | PC8 | 8th principal component score from functional principal component analysis (FPCA). |
|  | PC9 | 9th principal component score from functional principal component analysis (FPCA). |
|  | PC10 | 10th principal component score from functional principal component analysis (FPCA). |

**Supplementary Table 2.** The missing data for the classification tasks of significant anxiety symptoms.

| Accelerometer features on 7-day period | Missing proportion (%) | Accelerometer features on weekday | Missing proportion (%) |
| --- | --- | --- | --- |
| sleeponset | 1.46 | sleeponset | 1.95 |
| wakeup | 1.46 | wakeup | 1.95 |
| number_sib_wakinghours | 1.46 | number_sib_wakinghours | 1.95 |
| TST | 1.46 | TST | 1.95 |
| sleep_Midpoint | 1.46 | sleep_Midpoint | 1.95 |
| SE | 1.46 | SE | 1.95 |
| N_atleast5minwakenight | 1.95 | N_atleast5minwakenight | 2.93 |
| ACC_spt_sleep_mg | 1.95 | ACC_spt_sleep_mg | 2.93 |
| Nblocks_spt_sleep | 1.95 | Nblocks_spt_sleep | 2.93 |
| dur_day_total_IN_min | 0.49 | dur_day_total_IN_min | 0.98 |
| dur_day_total_LIG_min | 0.49 | dur_day_total_LIG_min | 0.98 |
| dur_day_total_MOD_min | 0.49 | dur_day_total_MOD_min | 0.98 |
| dur_day_total_VIG_min | 0.49 | dur_day_total_VIG_min | 0.98 |
| dur_day_MVPA_bts_10_min | 0.49 | dur_day_MVPA_bts_10_min | 0.98 |
| dur_day_MVPA_bts_5_10_min | 0.49 | dur_day_MVPA_bts_5_10_min | 0.98 |
| dur_day_MVPA_bts_1_5_min | 0.49 | dur_day_MVPA_bts_1_5_min | 0.98 |
| Nbouts_day_IN_bts_30 | 0.49 | Nbouts_day_IN_bts_30 | 0.98 |
| Nbouts_day_IN_bts_20_30 | 0.49 | Nbouts_day_IN_bts_20_30 | 0.98 |
| Nbouts_day_IN_bts_10_20 | 0.49 | Nbouts_day_IN_bts_10_20 | 0.98 |
| Nbouts_day_LIG_bts_10 | 0.49 | Nbouts_day_LIG_bts_10 | 0.98 |
| Nbouts_day_LIG_bts_5_10 | 0.49 | Nbouts_day_LIG_bts_5_10 | 0.98 |
| Nbouts_day_LIG_bts_1_5 | 0.49 | Nbouts_day_LIG_bts_1_5 | 0.98 |
| Nbouts_day_MVPA_bts_10 | 0.49 | Nbouts_day_MVPA_bts_10 | 0.98 |
| Nbouts_day_MVPA_bts_5_10 | 0.49 | Nbouts_day_MVPA_bts_5_10 | 0.98 |
| Nbouts_day_MVPA_bts_1_5 | 0.49 | Nbouts_day_MVPA_bts_1_5 | 0.98 |
| Nblocks_day_total_IN | 0.49 | Nblocks_day_total_IN | 0.98 |
| Nblocks_day_total_LIG | 0.49 | Nblocks_day_total_LIG | 0.98 |
| Nblocks_day_total_MOD | 0.49 | Nblocks_day_total_MOD | 0.98 |
| Nblocks_day_total_VIG | 0.49 | Nblocks_day_total_VIG | 0.98 |
| L5TIME_num | 0.49 | L5TIME_num | 0.98 |
| M10TIME_num | 0.49 | M10TIME_num | 0.98 |
| L5VALUE | 0.49 | L5VALUE | 0.98 |
| M10VALUE | 0.49 | M10VALUE | 0.98 |
| RA_ggir | 0.49 | RA_ggir | 0.98 |
|  |  |  |  |
|  |  |  |  |
|  |  |  |  |
|  |  |  |  |
|  |  |  |  |
| Accelerometer features on weekend | Proportion (%) | Accelerometer features on weekend | Proportion (%) |
| sleeponset | 2.93 | Nbouts_day_MVPA_bts_1_5 | 5.37 |
| wakeup | 2.93 | Nblocks_day_total_IN | 5.37 |
| number_sib_wakinghours | 2.93 | Nblocks_day_total_LIG | 5.37 |
| TST | 2.93 | Nblocks_day_total_MOD | 5.37 |
| sleep_Midpoint | 2.93 | Nblocks_day_total_VIG | 5.37 |
| SE | 2.93 | L5TIME_num | 5.37 |
| N_atleast5minwakenight | 7.32 | M10TIME_num | 5.37 |
| ACC_spt_sleep_mg | 7.32 | L5VALUE | 5.37 |
| Nblocks_spt_sleep | 7.32 | M10VALUE | 5.37 |
| TAC | 1.95 | RA_ggir | 5.37 |
| TLAC | 1.95 | L5 | 1.95 |
| TAC_24h | 1.95 | M10 | 1.95 |
| TLAC_24h | 1.95 | L5TIME | 1.95 |
| Sedentary time | 1.95 | M10TIME | 1.95 |
| Light activity time | 1.95 | RA | 1.95 |
| mod_dur | 1.95 | IV | 1.95 |
| vig_dur | 1.95 | IS_we | 1.46 |
| MVPA time | 1.95 | mesor_we | 1.95 |
| sed_dur_M10 | 1.95 | amp_we | 1.95 |
| light_dur_M10 | 1.95 | acro_we | 1.95 |
| mod_dur_M10 | 1.95 | acrotime_we | 1.95 |
| vig_dur_M10 | 1.95 | mesor_L_we | 1.95 |
| MVPA_dur_M10 | 1.95 | amp_L_we | 1.95 |
| sed_dur_24h | 1.95 | acro_L_we | 1.95 |
| light_dur_24h | 1.95 | acrotime_L_we | 1.95 |
| mod_dur_24h | 1.95 | minimum_ext_we | 1.95 |
| vig_dur_24h | 1.95 | amp_ext_we | 1.95 |
| MVPA_dur_24h | 1.95 | alpha_ext_we | 1.95 |
| mean_r | 1.95 | beta_ext_we | 1.95 |
| mean_a | 1.95 | acrotime_ext_we | 1.95 |
| SATP | 1.95 | F_pseudo_ext_we | 1.95 |
| ASTP | 1.95 | UpMesor_ext_we | 1.95 |
| Gini_r | 1.95 | DownMesor_ext_we | 1.95 |
| Gini_a | 1.95 | MESOR_ext_we | 1.95 |
| alpha_r | 1.95 | minimum_ext_L_we | 1.95 |
| alpha_a | 1.95 | amp_ext_L_we | 1.95 |
| h_r | 1.95 | alpha_ext_L_we | 1.95 |
| h_a | 1.95 | beta_ext_L_we | 1.95 |
| dur_day_total_IN_min | 5.37 | acrotime_ext_L_we | 1.95 |
| dur_day_total_LIG_min | 5.37 | F_pseudo_ext_L_we | 1.95 |
| dur_day_total_MOD_min | 5.37 | UpMesor_ext_L_we | 1.95 |
| dur_day_total_VIG_min | 5.37 | DownMesor_ext_L_we | 1.95 |
| dur_day_MVPA_bts_10_min | 5.37 | MESOR_ext_L_we | 1.95 |
| dur_day_MVPA_bts_5_10_min | 5.37 | PC1 | 1.95 |
| dur_day_MVPA_bts_1_5_min | 5.37 | PC2 | 1.95 |
| Nbouts_day_IN_bts_30 | 5.37 | PC3 | 1.95 |
| Nbouts_day_IN_bts_20_30 | 5.37 | PC4 | 1.95 |
| Nbouts_day_IN_bts_10_20 | 5.37 | PC5 | 1.95 |
| Nbouts_day_LIG_bts_10 | 5.37 | PC6 | 1.95 |
| Nbouts_day_LIG_bts_5_10 | 5.37 | PC7 | 1.95 |
| Nbouts_day_LIG_bts_1_5 | 5.37 | PC8 | 1.95 |
| Nbouts_day_MVPA_bts_10 | 5.37 | PC9 | 1.95 |
| Nbouts_day_MVPA_bts_5_10 | 5.37 | PC10 | 1.95 |

TST: total sleep time; SE: sleep efficiency; N_atleast5minwakenight: Number of times awake during the night for at least 5 minutes; ACC_spt_sleep_mg: Average acceleration during sleep (mg); Nblocks_spt_sleep: Number of blocks of night sleep within the Sleep period time window; TAC: Total volume of physical activity in the daytime; TLAC: Total volume of log-transformed physical activity in the daytime; TAC_24h: Total volume of physical activity within 24 hours; TLAC_24h: Total volume of log-transformed physical activity within 24 hours; mod_dur: Total daytime duration in minutes of moderate activity; vig_dur: Total daytime duration in minutes of vigorous activity ; MVPA：Moderate and Vigorous physical activity；sed_dur_M10：Total duration in minutes of sedentary activity within M10 window; light_dur_M10: Total duration in minutes of light activity within M10 window; mod_dur_M10: Total duration in minutes of moderate activity within M10 window; vig_dur_M10: Total duration in minutes of vigorous activity within M10 window; MVPA_dur_M10: Total duration in minutes of moderate to vigorous activity within M10 window; sed_dur_24h: Total duration in minutes of sedentary activity within 24 hours; light_dur_24h: Total duration in minutes of light activity within 24 hours; mod_dur_24h: Total duration in minutes of moderate activity within 24 hours; vig_dur_24h: Total duration in minutes of vigorous activity within 24 hours; MVPA_dur_24h: Total duration in minutes of moderate to vigorous activity within 24 hours; mean_a: Mean active bout duration; SATP: Sedentary to active transition probabilities; ASTP: Active to sedentary transition probabilities; Gini_r: Gini index for active bout; Gini_a: Gini index for sedentary bout; alpha_r: Power law parameter for sedentary bout; alpha_a: Power law parameter for active bout; h_r: Hazard function for sedentary bout; h_a: Hazard function for active bout; dur_day_total_IN_min: Total duration of day in minutes spent in total inactivity during the day; dur_day_total_LIG_min: Total duration of day in minutes of light activity during the day; dur_day_total_MOD_min: Total duration of day in minutes of moderate activity during the day; dur_day_total_VIG_min: Total duration of day in minutes of vigorous activity during the day; dur_day_MVPA_bts_10_min: Total duration in minutes of MVPA for bouts 10 minutes or more; dur_day_MVPA_bts_5_10_min: Total duration in minutes of MVPA for bouts 5 to 10 minutes; dur_day_MVPA_bts_1_5_min: Total duration in minutes of MVPA for bouts 1 to 5 minutes; Nbouts_day_IN_bts_30: Number of bouts of inactivity for bouts 30 minutes or more; Nbouts_day_IN_bts_20_30: Number of bouts of inactivity for bouts 20 to 30 minutes ; Nbouts_day_IN_bts_10_20: Number of bouts of inactivity for bouts 10 to 20 minutes ; Nbouts_day_LIG_bts_10: Number of bouts of light for bouts 10 minutes or more; Nbouts_day_LIG_bts_5_10: Number of bouts of light for bouts 5 to 10 minutes; Nbouts_day_LIG_bts_1_5: Number of bouts of light for bouts 1 to 5 minutes ; Nbouts_day_MVPA_bts_10: Number of bouts of MVPA for bouts 10 minutes or more; Nbouts_day_MVPA_bts_5_10: Number of bouts of MVPA for bouts 5 to 10 minutes; Nbouts_day_MVPA_bts_1_5: Number of bouts of MVPA for bouts 1 to 5 minutes; Nblocks_day_total_IN: Number of blocks of total inactivity during day; Nblocks_day_total_LIG: Number of blocks of total light activity during day; Nblocks_day_total_MOD: Number of blocks of total moderate activity during day; Nblocks_day_total_VIG: Number of blocks of total vigorous activity during day; L5TIME_num: Timing of least active 5 hours; M10TIME_num: Timing of most active 10 hours; L5VALUE: Average acceleration value (mg) of least active 5 hours; M10VALUE: Average acceleration value (mg) of most active 10 hours; RA_ggir: Relative amplitude reflects the difference between M10 activity and L5 activity; L5: Average acceleration value (mg) of least active 5 hours; M10: Average acceleration value (mg) of most active 10 hours; L5TIME: Timing of least active 5 hours; M10TIME: Timing of most active 10 hours; RA: Relative amplitude; IV: Intra-daily variability; IS: Inter-daily stability; amp: Amplitude; acro: Acrophase; mesor_L: Mesor in the cosinor model by using log-transformed data; amp_L: Amplitude log-transformed in the cosinor model by using log-transformed data; acro_L: Acrophase log-transformed in the cosinor model by using log-transformed data; PC1-PC10: 1^st^-10^th^ principal component score from functional principal component analysis.

**Supplementary Table 3.** Classification performance of diagnosing significant anxiety symptoms.

|  | **AUC** | **F1_Score** | **Youden** | **Sensitivity** | **Specificity** | **Accuracy** | **Recall** | **PPV** | **NPV** |
| --- | --- | --- | --- | --- | --- | --- | --- | --- | --- |
| **XGBoost-7d** | 0.713 (0.537,0.890) | 0.435 | 0.346 | 0.500 (0.187,0.813) | 0.846 (0.719,0.931) | 0.790 (0.668,0.883) | 0.500 (0.187,0.813) | 0.385 (0.139,0.684) | 0.898 (0.778,0.966) |
| **XGBoost-wd** | 0.777 (0.591,0.963) | 0.545 | 0.485 | 0.600 (0.262,0.878) | 0.885 (0.766,0.956) | 0.839 (0.723,0.920) | 0.600 (0.262,0.878) | 0.500 (0.211,0.789) | 0.920 (0.808,0.978) |
| **XGBoost-we** | 0.567 (0.381,0.754) | 0.211 | 0.065 | 0.200 (0.025,0.556) | 0.865 (0.742,0.944) | 0.758 (0.633,0.858) | 0.200 (0.025,0.556) | 0.222 (0.028,0.600) | 0.849 (0.724,0.933) |
| **XGBoost-merged** | 0.715 (0.569,0.862) | 0.222 | 0.085 | 0.200 (0.025,0.556) | 0.885 (0.766,0.956) | 0.774 (0.650,0.871) | 0.200 (0.025,0.556) | 0.250 (0.032,0.651) | 0.852 (0.729,0.934) |
| **DT-7d** | 0.633 (0.459,0.807) | 0.370 | 0.269 | 0.500 (0.187,0.813) | 0.769 (0.632,0.875) | 0.726 (0.598,0.831) | 0.500 (0.187,0.813) | 0.294 (0.103,0.560) | 0.889 (0.759,0.963) |
| **DT-wd** | 0.724 (0.546,0.902) | 0.480 | 0.427 | 0.600 (0.262,0.878) | 0.827 (0.697,0.918) | 0.790 (0.668,0.883) | 0.600 (0.262,0.878) | 0.400 (0.163,0.677) | 0.915 (0.796,0.976) |
| **DT-we** | 0.630 (0.448,0.812) | 0.400 | 0.308 | 0.500 (0.187,0.813) | 0.808 (0.675,0.904) | 0.758 (0.633,0.858) | 0.500 (0.187,0.813) | 0.333 (0.118,0.616) | 0.894 (0.769,0.965) |
| **DT-merged** | 0.735 (0.574,0.895) | 0.483 | 0.469 | 0.700 (0.348,0.933) | 0.769 (0.632,0.875) | 0.758 (0.633,0.858) | 0.700 (0.348,0.933) | 0.368 (0.163,0.616) | 0.930 (0.809,0.985) |
| **RF-7d** | 0.648 (0.434,0.863) | 0.400 | 0.285 | 0.400 (0.122,0.738) | 0.885 (0.766,0.956) | 0.806 (0.686,0.896) | 0.400 (0.122,0.738) | 0.400 (0.122,0.738) | 0.885 (0.766,0.956) |
| **RF-wd** | 0.712 (0.532,0.891) | 0.417 | 0.327 | 0.500 (0.187,0.813) | 0.827 (0.697,0.918) | 0.774 (0.650,0.871) | 0.500 (0.187,0.813) | 0.357 (0.128,0.649) | 0.896 (0.773,0.965) |
| **RF-we** | 0.631 (0.473,0.789) | 0.211 | 0.065 | 0.200 (0.025,0.556) | 0.865 (0.742,0.944) | 0.758 (0.633,0.858) | 0.200 (0.025,0.556) | 0.222 (0.028,0.600) | 0.849 (0.724,0.933) |
| **RF-merged** | 0.690 (0.506,0.875) | 0.133 | 0.023 | 0.100 (0.003,0.445) | 0.923 (0.815,0.979) | 0.790 (0.668,0.883) | 0.100 (0.003,0.445) | 0.200 (0.005,0.716) | 0.842 (0.721,0.925) |
| **SVM-7d** | 0.548 (0.323,0.774) | 0.222 | 0.031 | 0.300 (0.067,0.652) | 0.731 (0.590,0.844) | 0.661 (0.530,0.777) | 0.300 (0.067,0.652) | 0.176 (0.038,0.434) | 0.844 (0.705,0.935) |
| **SVM-wd** | 0.631 (0.413,0.849) | 0.345 | 0.231 | 0.500 (0.187,0.813) | 0.731 (0.590,0.844) | 0.694 (0.563,0.804) | 0.500 (0.187,0.813) | 0.263 (0.091,0.512) | 0.884 (0.749,0.961) |
| **SVM-we** | 0.527 (0.321,0.733) | 0.258 | 0.073 | 0.400 (0.122,0.738) | 0.673 (0.529,0.797) | 0.629 (0.497,0.748) | 0.400 (0.122,0.738) | 0.190 (0.054,0.419) | 0.854 (0.708,0.944) |
| **SVM-merged** | 0.519 (0.313,0.726) | 0.091 | -0.112 | 0.100 (0.003,0.445) | 0.788 (0.653,0.889) | 0.677 (0.547,0.791) | 0.100 (0.003,0.445) | 0.083 (0.002,0.385) | 0.820 (0.686,0.914) |
| **KNN-7d** | 0.712 (0.501,0.921) | 0.410 | 0.396 | 0.800 (0.444,0.975) | 0.596 (0.451,0.730) | 0.629 (0.497,0.748) | 0.800 (0.444,0.975) | 0.276 (0.127,0.472) | 0.939 (0.798,0.993) |
| **KNN-wd** | 0.726 (0.557,0.895) | 0.410 | 0.396 | 0.800 (0.444,0.975) | 0.596 (0.451,0.730) | 0.629 (0.497,0.748) | 0.800 (0.444,0.975) | 0.276 (0.127,0.472) | 0.939 (0.798,0.993) |
| **KNN-we** | 0.526 (0.319,0.733) | 0.211 | -0.062 | 0.400 (0.122,0.738) | 0.538 (0.395,0.678) | 0.516 (0.386,0.645) | 0.400 (0.122,0.738) | 0.143 (0.040,0.327) | 0.824 (0.655,0.932) |
| **KNN-merged** | 0.556 (0.361,0.751) | 0.286 | 0.100 | 0.600 (0.262,0.878) | 0.500 (0.358,0.642) | 0.516 (0.386,0.645) | 0.600 (0.262,0.878) | 0.188 (0.072,0.364) | 0.867 (0.693,0.962) |
| **Adaboost-7d** | 0.640 (0.463,0.818) | 0.190 | 0.027 | 0.200 (0.025,0.556) | 0.827 (0.697,0.918) | 0.726 (0.598,0.831) | 0.200 (0.025,0.556) | 0.182 (0.023,0.518) | 0.843 (0.714,0.930) |
| **Adaboost-wd** | 0.715 (0.538,0.893) | 0.417 | 0.327 | 0.500 (0.187,0.813) | 0.827 (0.697,0.918) | 0.774 (0.650,0.871) | 0.500 (0.187,0.813) | 0.357 (0.128,0.649) | 0.896 (0.773,0.965) |
| **Adaboost-we** | 0.656 (0.476,0.835) | 0.222 | 0.085 | 0.200 (0.025,0.556) | 0.885 (0.766,0.956) | 0.774 (0.650,0.871) | 0.200 (0.025,0.556) | 0.250 (0.032,0.651) | 0.852 (0.729,0.934) |
| **Adaboost-merged** | 0.619 (0.433,0.805) | 0.118 | -0.015 | 0.100 (0.003,0.445) | 0.885 (0.766,0.956) | 0.758 (0.633,0.858) | 0.100 (0.003,0.445) | 0.143 (0.004,0.579) | 0.836 (0.712,0.922) |
| **MLP-7d** | 0.498 (0.284,0.712) | 0.286 | 0.146 | 0.300 (0.067,0.652) | 0.846 (0.719,0.931) | 0.758 (0.633,0.858) | 0.300 (0.067,0.652) | 0.273 (0.060,0.610) | 0.863 (0.737,0.943) |
| **MLP-wd** | 0.769 (0.629,0.909) | 0.385 | 0.288 | 0.500 (0.187,0.813) | 0.788 (0.653,0.889) | 0.742 (0.615,0.845) | 0.500 (0.187,0.813) | 0.312 (0.110,0.587) | 0.891 (0.764,0.964) |
| **MLP-we** | 0.617 (0.420,0.815) | 0.105 | -0.054 | 0.100 (0.003,0.445) | 0.846 (0.719,0.931) | 0.726 (0.598,0.831) | 0.100 (0.003,0.445) | 0.111 (0.003,0.482) | 0.830 (0.702,0.919) |
| **MLP-merged** | 0.569 (0.362,0.777) | 0.19 | 0.027 | 0.200 (0.025,0.556) | 0.827 (0.697,0.918) | 0.726 (0.598,0.831) | 0.200 (0.025,0.556) | 0.182 (0.023,0.518) | 0.843 (0.714,0.930) |
| **NB-7d** | 0.672 (0.464,0.880) | 0.414 | 0.35 | 0.600 (0.262,0.878) | 0.750 (0.611,0.860) | 0.726 (0.598,0.831) | 0.600 (0.262,0.878) | 0.316 (0.126,0.566) | 0.907 (0.779,0.974) |
| **NB-wd** | 0.587 (0.354,0.820) | 0.32 | 0.188 | 0.400 (0.122,0.738) | 0.788 (0.653,0.889) | 0.726 (0.598,0.831) | 0.400 (0.122,0.738) | 0.267 (0.078,0.551) | 0.872 (0.743,0.952) |
| **NB-we** | 0.515 (0.280,0.750) | 0.263 | 0.058 | 0.500 (0.187,0.813) | 0.558 (0.413,0.695) | 0.548 (0.417,0.675) | 0.500 (0.187,0.813) | 0.179 (0.061,0.369) | 0.853 (0.689,0.950) |
| **NB-merged** | 0.502 (0.265,0.739) | 0.222 | 0.031 | 0.300 (0.067,0.652) | 0.731 (0.590,0.844) | 0.661 (0.530,0.777) | 0.300 (0.067,0.652) | 0.176 (0.038,0.434) | 0.844 (0.705,0.935) |

Abbrevation: XGBoost: extreme gradient boosting; DT: decision tree; RF: random forest; SVM: support vector machine; KNN: k-nearest neighbor; Adaboost: adaptive boosting; MLP: multilayer perceptron; NB: naive bayes; wd: weekday; we: weekend..
